# Supplementary material for: Overcoming barriers to engagement and adherence to a home-based physical activity intervention for patients with heart failure: a qualitative focus group study
Source: BMJ Open. 2020 Sep 21;10(9):e036382. doi: 10.1136/bmjopen-2019-036382 (PMC7507843; doi:10.1136/bmjopen-2019-036382)
Supplement: Author's manuscript [file bmjopen-2019-036382.draft_revisions.pdf]

BMJ Open is committed to open peer review. As part of this commitment we make the peer review history of every article we publish publicly available.

When an article is published we post the peer reviewers' comments and the authors' responses online. We also post the versions of the paper that were used during peer review. These are the versions that the peer review comments apply to.

The versions of the paper that follow are the versions that were submitted during the peer review process. They are not the versions of record or the final published versions. They should not be cited or distributed as the published version of this manuscript.

BMJ Open is an open access journal and the full, final, typeset and author-corrected version of record of the manuscript is available on our site with no access controls, subscription charges or pay-per-view fees (http://bmjopen.bmj.com).

If you have any questions on BMJ Open's open peer review process please email info.bmjopen@bmj.com

BMJ Open

Overcoming barriers to engagement and adherence to a home-based physical activity intervention for patients with heart failure: A qualitative focus group study

Journal:	*BMJ Open*
Manuscript ID	bmjopen-2019-036382
Article Type:	Original research
Date Submitted by the Author:	21-Dec-2019

Complete List of Authors:	Okwose, Nduka; Newcastle University Faculty of Medical Sciences, Translational and Clinical Research Institute<br>O'Brien, Nicola; Northumbria University - City Campus, Department of Psychology<br>Charman, Sarah; Newcastle University Faculty of Medical Sciences, Translational and Clinical Research Institute<br>Cassidy, Sophie; Newcastle University Faculty of Medical Sciences, Translational and Clinical Research Institute<br>Brodie, David; Buckinghamshire New University, School of Health and Social Sciences<br>Bailey, Kristian; Newcastle upon Tyne Hospitals NHS Foundation Trust<br>MacGowan, Guy; Newcastle Upon Tyne Hospitals NHS Foundation Trust, Cardiology<br>Jakovljevic, Djordje; Newcastle University Faculty of Medical Sciences, Translational and Clinical Research Institute<br>Avery, Leah; Teesside University, School of Health & Life Sciences

Keywords:	Heart failure < CARDIOLOGY, QUALITATIVE RESEARCH, Adult cardiology < CARDIOLOGY

SCHOLARONE™
Manuscripts

BMJ

I, the Submitting Author has the right to grant and does grant on behalf of all authors of the Work (as defined in the below author licence), an exclusive licence and/or a non-exclusive licence for contributions from authors who are: i) UK Crown employees; ii) where BMJ has agreed a CC-BY licence shall apply, and/or iii) in accordance with the terms applicable for US Federal Government officers or employees acting as part of their official duties; on a worldwide, perpetual, irrevocable, royalty-free basis to BMJ Publishing Group Ltd ("BMJ") its licensees and where the relevant Journal is co-owned by BMJ to the co-owners of the Journal, to publish the Work in this journal and any other BMJ products and to exploit all rights, as set out in our licence.

The Submitting Author accepts and understands that any supply made under these terms is made by BMJ to the Submitting Author unless you are acting as an employee on behalf of your employer or a postgraduate student of an affiliated institution which is paying any applicable article publishing charge ("APC") for Open Access articles. Where the Submitting Author wishes to make the Work available on an Open Access basis (and intends to pay the relevant APC), the terms of reuse of such Open Access shall be governed by a Creative Commons licence – details of these licences and which Creative Commons licence will apply to this Work are set out in our licence referred to above.

Other than as permitted in any relevant BMJ Author's Self Archiving Policies, I confirm this Work has not been accepted for publication elsewhere, is not being considered for publication elsewhere and does not duplicate material already published. I confirm all authors consent to publication of this Work and authorise the granting of this licence.

For peer review only

Overcoming barriers to engagement and adherence to a home-based physical activity intervention for patients with heart failure: A qualitative focus group study

Nduka C Okwose, PhD[1] , Nicola O'Brien, PhD[2,3], Sarah J Charman, PhD[1], Sophie Cassidy, PhD[1], David A Brodie[4] DSci, Kristian Bailey, MD[5] , Guy A MacGowan, MD[5,6] , Djordje G Jakovljevic, PhD[1,5,] *Leah Avery, PhD[2,6]

[1] Cardiovascular Research Centre; Translational and Clinical Research Institute, Faculty of Medical Sciences, Newcastle University, UK

[2]Population Health Sciences Research Institute, Faculty of Medical Sciences, Newcastle University, UK

[3] Department of Psychology, Northumbria University, UK

[4] School of Health and Social Sciences, Buckinghamshire New University, Uxbridge, UK

[5] Newcastle upon Tyne Hospitals NHS Foundation Trust, Newcastle upon Tyne, UK

[6]Biosciences Institute, Faculty of Medical Sciences, Newcastle University, UK

[6] School of Health and Life Sciences, Teesside University, Tees Valley, UK

Corresponding Author: Dr Leah Avery: leah.avery@tees.ac.uk, Centre for Rehabilitation, Exercise and Sports Sciences, School of Health and Life Sciences, Teesside University, Tees Valley, UK. TS1 3BA. Tel: +44 (0)1642 384130; Email: leah.avery@tees.ac.uk.

For peer review only

Conflict of interest statement: This research was funded by the National Institute of Health Research (NIHR). The funder and study sponsor played no role in the study design; collection, analysis and interpretation of data; writing the manuscript; or the decision to submit the manuscript for publication. The authors declare that they have no conflicts of interest.

Abstract

Introduction: Clinical guidelines recommend regular physical activity for patients with heart failure to improve functional capacity and symptoms and to reduce hospitalisation. Cardiac rehabilitation programmes have demonstrated success in this regard, however uptake and adherence are sub-optimal. Home-based physical activity programmes have gained popularity to address these issues, although it is acknowledged that proximity of programmes and individual needs and preferences of patients are likely to impact upon their effectiveness. The study aimed to identify barriers and facilitators to engagement and adherence to a home-based physical activity programme, and to identify ways in which it could be integrated into the care pathway for patients with heart failure.

Methods: Three focus group discussions were conducted with 16 patients (82% males, aged: 68 ± 7 years; heart failure duration ≥9 years) who were either taking part in a home-based physical activity intervention or who declined participation. Data were analysed using thematic analysis.

Results: Ten main themes were generated that provided a comprehensive overview of what the active ingredients of the intervention were in terms of engagement and continued participation. Fear of engaging in physical activity was reported to be a significant barrier to engagement. Influences of family members were both barriers and facilitators to engagement and adherence. Facilitators included endorsement of physical activity by clinicians

For peer review only

knowledgeable in physical activity in the context of heart failure; ongoing support and personalised feedback from trained team members; and tailoring to meet individual needs, overcome barriers and increase confidence.

Conclusions: Endorsement of home-based programmes by the clinical team to reduce patients' fear of undertaking physical activity and individual tailoring to overcome barriers are necessary for long-term adherence. Family members should be encouraged to attend consultations to address misconceptions and fear about the contraindications of physical activity in the context of heart failure.

Strengths and limitations of this study

- Qualitative focus groups are an appropriate methodological approach to explore views on uptake and engagement with an intervention and can help facilitate consensus
- Adults with heart failure who had declined participation in the intervention and those who had previously attended centre-based programmes, as well as those who had not were recruited to obtain representativeness of views
- A higher proportion of men participated, therefore the views of women should be explored further
- The outcome of the methodological approach undertaken will inform intervention optimisation

Introduction

Increasing levels of physical activity is recommended to improve cardio-metabolic outcomes and to enhance overall health and psychological wellbeing[1–3]. In the UK, cardiac

For peer review only

rehabilitation is offered as a pathway of referral for people living with chronic conditions such as cardiovascular disease. Encouraging patients to attend is often challenging, although uptake increased from 45% in 2013 to 51% in 2016[4]. However, these figures provide an artificial estimate of attendance because only 5.3% of patients with heart failure actually attend cardiac rehabilitation[4]. Furthermore, dropout rates often exceed 20% for those attending centre-based programmes[5]. Barriers to uptake and adherence are reported to include proximity of cardiac rehabilitation services and motivation levels of patients to attend[5,6].

A potential solution to overcome these barriers is the provision of home-based physical activity programmes. Home-based programmes have shown to elicit similar outcomes to centre-based programmes and could be particularly attractive to those with a preference to exercise at home. They also have the potential to be tailored to individual needs and preferences, thereby increasing engagement with the programme and reducing dropout rates[7,8].

This qualitative study explored the barriers and facilitators to engagement and adherence to a home-based physical activity programme for patients with heart failure, called 'Active-at-Home-HF'.

Methods

This study was approved by the NHS North East – Tyne and Wear South Research Ethics Committee (REC reference: 15/NE/0190). All participants provided informed consent prior to participation.

Intervention

Active-at-Home-HF is a behavioural intervention designed to support patients with heart failure to increase their baseline physical activity levels by at least 2000 steps per day over a period of 12 weeks. Three researchers with backgrounds in exercise physiology were trained by a Chartered Health Psychologist to deliver the intervention adopting the role of a lifestyle

For peer review only

coach. Coaches used evidence-based behaviour change techniques including behavioural goal setting, action planning, problem solving, self-monitoring, feedback on behaviour and review of behavioural goals [10, 11, 12]. Participants were supported to increase and sustain physical activity levels via telephone calls lasting between 8 and 15 minutes on at least 10 occasions, i.e. weekly over a period of 12 weeks. The number of telephone calls was dictated by the participant up to a maximum of 12 calls (i.e. one call per week). Physical activity goals were set and modified in collaboration with the coach and participant depending upon each participant's progress, capability, needs and preferences, with an emphasis on duration of physical activity rather than intensity. A standardised proforma was used to structure calls and to serve as a prompt to use all behaviour change techniques, where appropriate and to increase fidelity of delivery. All three researchers who delivered the intervention also completed study visits with partipants during the Active-at-Home-HF pilot study.

Design

A qualitative study embedded within a pilot study of the Active-at-Home-HF intervention was conducted [13]. Focus group discussions were used to identify barriers and facilitators to intervention uptake and adherence, and ways in which the intervention could be integrated into routine clinical care were explored.

Participants

Eligible participants had left ventricular ejection fraction ≤ 40% diagnosed at least three months previously; were clinically stable and receiving an optimal medical treatment; had no contraindications to physical activity; and were able to walk and perform activities of daily living independently. The aim was to recruit a purposive sample of between 12 and 20 participants in line with published guidance and previously published qualitative research to ensure data saturation[9,10]. Eligible participants included those who had previously taken part

For peer review only

in a centre-based cardiac rehabilitation programme to ensure a representation of views and experiences, and also included those who had refused participation in Active-at-Home-HF to identify barriers to uptake. Participants were contacted via email, telephone, or spoken to in person to discuss participation in the study and given an opportunity to ask questions to ensure they understood the aims of the study.

Study Procedure

Eligible participants under the care of a cardiologist at the Freeman Hospital, Newcastle upon Tyne, UK were invited to take part in the study. Focus group discussions were facilitated by two female Chartered Health Psychologists with expertise in health behaviour change, physical activity intervention and qualitative research methods. Neither of the researchers had met the participants prior to the focus group discussions and neither were aware of any preliminary findings of the Active-at-Home-HF pilot study. The focus group discussions were conducted in a meeting room at the Royal Victoria Infirmary, Newcastle upon Tyne. Only the two researchers and the participants were present during the focus group discussions.

Materials

The research team developed a topic guide to structure discussions. Topics including reasons for taking part, or declining participation; preference for completing a home-based programme; information required to inform decision-making regarding participation; support requirements to complete the programme and potential barriers; and expectations of the programme. All questions were open-ended and prompts were used to generate a deeper understanding of participants' views. Each focus group discussion was audio recorded and transcribed verbatim.

Methodological Quality and reporting

The research was conducted in accordance with the consolidated criteria for reporting qualitative research (COREQ) to enhance transparency and maximise methodological

For peer review only

quality[11]. To reduce bias from responders, members of the team delivering the intervention or involved in the pilot study clinic visits were not involved with focus group discussions.

Data Analysis

Data were analysed using thematic analysis[12]. To maximise methodological quality and trustworthiness of the findings, the following analysis procedure was undertaken: (i) Two researchers independently read and re-read focus group transcripts (NCO and LA); (ii) both researchers independently applied codes to segments of data within the first focus group transcript to develop initial themes and sub-themes; (iii) the same two researchers discussed findings and agreed a preliminary set of themes and sub-themes; (iv) one researcher (NCO) repeated this process with the remaining two transcripts; and (v) both researchers agreed the final set of themes and sub-themes that adequately represented the data set following discussion. Supporting direct quotes from participants were subsequently applied to themes and sub-themes. A third researcher (NO'B) reviewed themes and sub-themes generated to verify interpretation.

Patient and public involvement

There was no patient and public involvement in the design or planning of the study.

Results

Three focus groups were conducted, with a duration of 47, 53 and 83 minutes respectively. A total of sixteen individuals (13 males, 3 females; median age 68 years; interquartile range: 6.5 years) from the twenty who were eligible to take part in the pilot study participated in the focus group discussions. Participants had either completed Active-at-Home-HF (4 participants), were still participating at the time of the focus group (10 participants), or had declined participation (2 participants). Ten participants had implanted cardiac defibrillators (ICDs). Fifteen of the

For peer review only

sixteen participants were retired from active work. Following thematic analyses, 10 main themes were generated. It was noted following the conduct of focus group three that no new data was generated, i.e. it was agreed that data saturation was reached. Table 1 provides a summary of the themes and subthemes, and example direct quotes to provide context.

For peer review only

Table 1: A summary of themes and subthemes derived from thematic analyses of focus group discussion transcripts

Theme	Sub-theme	Quotes
**1. Fear of undertaking physical activity**		'I'm not confident when I go out on my own. I'm frightened I'm going to collapse' (FG 1, Male, aged 57)<br>'It's frightening when you've gone too far. Something's always holding me back from going that little bit further, you know' (FG 1, Male, aged 56)
**2. Family members influence physical activity efforts**	**2.1 Fear in family members prevents engaging with physical activity**	'I've got a wife who's not happy if I say I'm going out for a walk for an hour.., because she thinks that's too much' (FG 2, Male, aged 67)<br>'..Sometimes, your family don't want you to do it, not because they're being horrible, it's because they're concerned about you' (FG 1, Male, aged 78)
	**2.2 Support from family members facilitates physical activity**	'I needed motivation which I got from the family, which is great…I don't know how I would have fared without family' (FG 2, Female, aged 62)<br>'I think it does help if you've got a supportive partner. My husband has come out with me for all my walks right from the start. I think it's good if your partners can be involved and then they realise, don't they, how they can help' (FG 2, Female, aged 62)
**3. Physical activity programmes require endorsement by clinicians**		'I think my biggest breakthrough was . . . I was doing more and more but, again, family was saying, "Take it easy." Fortunately, my wife was with me on one occasion when I saw Dr "XX" and he asked what I'd been doing, and he said, "Well, do more." I said,

For peer review only

	"How do you mean by that?" He said, "Just do more and more until you feel you can't and then back off a bit." So, fortunately, my wife was there and she heard him say it. So, I've had no peace since then' (FG 1, Male, aged 78) <br>'My nurse certainly was interested [in my physical activity habits] and when I went last time, was aware of what I was doing and obviously encouraging' (FG 2, Female, aged 62)
**4. Completion of a cardiac rehabilitation programme provides confidence to complete a physical activity programme at home**	'I think the most important thing when you've had a heart attack is to have the confidence to do it at all. I think the cardiac rehabilitation that I had gave me the confidence and then you can move on to do other things. I think if you had just had a heart attack and someone says, "Right, you've got to go for a walk every day," it would be difficult to do. I think it would. So it's like a step in the process' (FG 2, Male, aged 71) <br>'I was really upset when it [cardiac rehabilitation] finished. I had somewhere to go. I'm sat at home by myself all day, so coming here on a Wednesday, it was like Christmas all over again, you know. It was great. (FG 1, Male, aged 56)
**5. Coach support increases motivation long-term**	'[having your coach call you] encourages you to keep up with it, definitely, because I think if you didn't get the phone calls, I think you might just go, "It's not very nice out today, I'll not bother going'(FG 2, Female, aged 62)

For peer review only

	‘When somebody is monitoring you, it makes you get up and go out, doesn't it? I mean if you go to the gym yourself, some mornings you might say, "I'm not going to go today," but it's helping you, isn't it?’ (FG 3, Male, aged 62)
**6. Weekly agreed targets increased confidence and motivation**	‘It's a target as everyone's saying and you want to do it and you feel very enthusiastic about doing it and I will certainly continue after the 12 weeks because it would be pointless stopping all together wouldn't it? It would waste the benefit sort of thing’ (FG 2, Male, aged 70)<br><br>‘I think if you were told increase to 12,000 and I come back in 12 weeks’ time and we talk about it, you would go... but what's good is on Tuesday [my coach] rings me and goes through all the information, we get an average and then he'll ask me how I'm feeling, etc., etc., and say, "Right, let's try and take that 12,000 up to 12,500." That's on a weekly basis. You know for a fact that someone's interested in what's been happening for the past week and we can take that from where we are now to try and improve things.’ (FG 2, Male, aged 69)
**7. A credible team increased the likelihood of participation**	‘I think the fact that people like yourselves who are specialists in this sort of area take such an interest in us people. I think that gives you the boost again. I think it does boost people when you've got people who are really even higher than your GP and what not in that specific area of cardiac problems’ (FG 2, Male, aged 78)

For peer review only

	‘Well I think this is a speciality subject, what you're doing and all the stuff here that we've been doing. I think it's better in that situation to get the right advice’ (FG 2, Male, aged 67)
**8. The surrounding environment creates barriers to increased physical activity/exercise**	‘I think another …. difficulty… trying to get people motivated is the area that they live in. Now if you've got a bit of countryside, open fields and that, it opens you to more space unlike just got solid concrete, trying to get people motivated to walk down the same street or go a particular… way, that’s going to be difficult I think. If you're going to motivate people you're going to have to think where they actually live because people need different types of motivation. For people who live near the coast and the countryside, people living smack bang in the middle of a built-up area etc’ (FG 2, Male, aged 67)<br><br>‘To me, I’m okay most of the time on the flat, but it’s any incline. The littlest incline in the world kills me’ (FG 2, Male, aged 78)<br><br>‘I think some of the benefit was the climate as well, it’s a struggle in wind and rain’ (FG 3, Female, aged 63)
**9. Participation prompts an increase in every day activity levels**	‘What I do now is I have to drive to the supermarket but I park in the furthest corner of the car park and I walk round the car park. Then on rainy days, what I have been doing is going into the supermarket and going round it twice before I start my shopping. The people must think you're mad if they look at you on the CCTV’ (FG 2, Male, aged 71)

For peer review only

		'I think it's got to work doing exercise. I mean they brought me in here; they're going on about putting a defibrillator in. Now when he saw me, how my heart had changed just through doing things, he decided he wouldn't put one in' (FG 3, Male, aged 62)<br><br>'I haven't been back to see my specialist at the Freeman [hospital], because he doesn't want to see me for 12 months because I'm too fit for him. I said, "Are you sick of me?" he said, "No." It was six months before and then I went for the last check sometime last year, he said, "Right, I don't want to see you for 12 months. I said, have you gone off me?' (FG 2, Male, aged 71)<br><br>'Well since I've completed the course [Active-at-Home-HF], I've been diabetic for 30 years, my blood sugar levels have never been as normal as an ordinary person's in my life' (FG 2, Male, aged 79)
**10. Support to maintain long-term activity levels would be beneficial**	**10.1 Support from a healthcare professional following completion of the programme would help maintain increased physical activity**	'I think a follow-up is a good idea. You need some sort of follow-up after you've finished. How they do it, whether it's a phone call or a meeting with your doctor or whatever, your GP or anybody like that, I don't know how they would do it. But I think that's quite important that. Then you wouldn't feel as if you've been chucked on the scrap heap type of thing, you've finished, it's done' (FG 3, Male, aged 73)<br><br>'Do you know, that's the worst thing, when you've finished your course and the following Wednesday there's no phone call. It's horrendous isn't it?' (FG 2, Male, aged 67)

For peer review only

**10.2 Group social support would support long-term physical activity**	'I know it's just the start of this programme …, As far as feedback on it is concerned, maybe something about halfway through, maybe six weeks you could have a little meeting with some of the people just for half an hour, just have a little chat and see who's there, what's what, talk to people, something like that anyway. A little informal meeting or a social evening or whatever you want to call it. It lets everybody else know that it's not just you or another two or three people, it might be 20 people' (FG 3, Male, aged 73)

For peer review only

Fear of undertaking physical activity

Once diagnosed with heart failure, participants reported difficulties overcoming the fear associated with the diagnosis (i.e. future cardiac events and overexertion) and increasing physical activity exacerbated this fear. While some reported that they could still engage in some physical activity, there was concern that too much would be detrimental. This was reported to be a significant barrier to participation: *'It's frightening when you've gone too far. Something's always holding me back from going that little bit further, you know'* (FG 1, Male, aged 56). In this regard, it was reported that reassurance from a clinican is required to confirm that increasing physical activity is safe, and specific information in terms of how much activity should be undertaken to be beneficial, and not detrimental.

Family members influence engagement with physical activity

It was reported that family members were both a barrier and facilitator to engaging in physical activity. Some participants reported that family members dissuaded them from engaging in physical activity. Although, there was a consensus that this was linked to fear and concern *'Sometimes, your family don't want you to do it, not because they're being horrible, it's because they're concerned about you'* (FG 2, Male, aged 78). Fear of family members often meant that participants were less active following a diagnosis of heart failure than they were previously.

It was also reported that family members were a strong motivating factor once they were reassured that increasing physical activity was beneficial. For some participants, having a partner or relative to support them and participate in new activity routines was reported as a significant facilitator to continued participation. A typical quote included: *'I think it does help if you've got a supportive partner. My husband has come out with me for all my walks right*

For peer review only

from the start. I think it's good if your partners can be involved and then they realise, don't they, how they can help' (FG 2, Female, aged 62). There was a consensus that without the practical and emotional support of partners and relatives, increasing physical activity would be more difficult.

Physical activity programmes require endorsement by clinicians

Endorsement of the physical activity programmes by a clinician was reported to reduce patients' fears about increasing levels of physical activity and potentially making their condition worse. Furthermore, it reassured family members when they witnessed clinicians explaining the benefits and advising an increase. In addition, ongoing positive reinforcement by the clinical team was considered paramount to continued participation: '*My nurse certainly was interested [in my physical activity levels] and when I went last time, was aware of what I was doing and was encouraging'* (FG 2, Female, aged 62).

Prior completion of a cardiac rehabilitation programme provides confidence to engage with home-based physical activity programmes

Cardiac rehabilitation was reported to play a significant role in confidence building among heart failure patients, including confidence to continue with activities of daily living. As such it was reported to be a significant facilitator to uptake of Active-At-Home-HF as a means of providing patients with ongoing support and encouragement to be physically active in the long-term. Continued participation was particularly important to those who had completed a cardiac rehabilitation programme: *'I was really upset when it [cardiac rehabilitation] finished. I had somewhere to go. I'm sat at home by myself all day, so coming here on a Wednesday, it was like Christmas all over again, you know. It was great'* (FG 1, Male, aged 56).

For peer review only

Behavioural support provided by a trained professional increases motivation and adherence

Participants reported that having a trained professional who monitored their performance and provided tailored advice and support promoted continued participation: *'[having your coach call you] encourages you to keep up with it, definitely, because I think if you didn't get the phone calls, I think you might just go, "It's not very nice out today, I'll not bother going'*(FG 2, Female, aged 62). It was important that the support was provided by someone who could advise on the type, amount and duration of activity required to reach a specific target; and someone who was skilled in the use of behavioural strategies who could appropriately challenge participants and support them to overcome perceived barriers.

Agreed weekly targets increased confidence and motivation

Physical activity goal setting was considered an important mechanism to increase motivation, particularly when weekly goals were personalised and agreed between the participant and coach. This provided reassurance that goals were realistic, attainable and safe and it was reported to be rewarding when goals were achieved. Some participants were keen to set themselves daily or weekly targets once they had knowledge and confidence in their capabilities, highlighting the potential long-term impact of the programme.

> *'It's a target as everyone's saying and you want to do it and you feel very enthusiastic about doing it and I will certainly continue after the 12 weeks because it would be pointless stopping all together wouldn't it?'* (FG 2, male, aged 70).

For peer review only

A credible team increases the likelihood of participation

An incentive of taking part in Active-at-Home-HF was reported to be the support and monitoring offered by experts in heart failure and physical activity. Participants emphasised that endorsement of the programme by a Consultant who was knowledgeable about the benefits of physical activity in the context of heart failure, and a referral to a trained professional/member of his or her team who was aware of their clinical circumstances helped overcome barriers to ongoing participation. The 'knowledgeable team' provided reassurance around safety, and was skilled in tailoring the intervention to individual needs and preferences and this was considered important to facilitate initial engagement and long-term participation.

> *'Well I think this is a speciality subject, what you're doing and all the stuff here know what we've been doing. I think it's better in that situation to get the right advice'* (FG 2, Male, aged 67).

The surrounding environment creates barriers to increasing physical activity and maintaining a physically active lifestyle

Environmental factors including living in hilly areas or in surroundings that were not considered picturesque were reported to be barriers to engagement and ongoing participation in physical activity. Poor weather conditions were most frequently reported to impact negatively on motivation and long-term participation.

> *'To me, I'm okay most of the time on the flat, but it's any incline. The littlest incline in the world kills me'* (FG 2, Male, aged 78).

For peer review only

'I think some of the benefit was the climate as well, because it was through the summer' (FG 3, Female, aged 63).

Participants reported that the support they received via Active-at-Home-HF enabled them to identify ways in which they could increase their levels of activity despite these barriers, for example planning alternative ways to increase their step count and reach their overall goals. In this regard the programme increased motivation and realisation that there was an alternative approach to increasing activity to a level sufficient to have a clinically meaningful impact.

Participation prompts an increase in every day activity levels and leads to unexpected positive outcomes

Active-at-Home-HF was reported to encourage participants to seek ways to increase their activity levels by modifying their everyday behaviours (e.g. walking instead of driving). One participant reported how the intervention prompted him to spend more time with his grandchildren, thereby positively affecting activity levels and social connections with his family. Some described how their fitness and feeling of wellbeing had improved (this was confirmed in the pilot study) and the frequency of appointments with their consultant had reduced following progress.

'I haven't been back to see my specialist at the hospital, because he doesn't want to see me for 12 months because I'm too fit for him. I said, "Are you sick of me?" he said, "No." It was six months before and then I went for the last check sometime last year, he said, "Right, I don't want to see you for 12 months' (FG 2, male, aged 71).

The intervention led to improvements in cardiac function for the majority of those who participated. For some, this outcome meant avoiding invasive procedures that were planned

For peer review only

prior to the study. For other participants the intervention helped to improve cardiac function and to control other co-morbidities.

> *'Well since I've completed the course [Active-at-Home-HF], I've been diabetic for 30 years, my blood sugar levels have never been as normal as an ordinary person's in my life'* (FG 2, male, aged 79).

Ongoing support would facilitate long-term maintenance of physical activity

Continued support was considered critically important for psychological and emotional wellbeing as well as maintenance of activity levels. It was emphasised that support should include a review of progress to promote ongoing motivation.

> *'Do you know, that's the worst thing, when you've finished your course and the following Wednesday there's no phone call. It's horrendous isn't it?'* (FG 2, male, aged 67).

There was a general consensus that having frequent meetings with others using the programme would be beneficial for social support and promoting increased physical activity levels long-term.

> *'Maybe something about halfway through, maybe six weeks you could have a little meeting with some of the people just for half an hour, just have a little chat and see who's there, what's what, talk to people, something like that anyway. A little informal meeting or a social evening or whatever you want to call it. It lets everybody else know that it's not just you or another two or three people, it might be 20 people'* (FG 3, male, aged 73).

For peer review only

A consistent theme across focus group discussions was the need for ongoing support to maintain physical activity levels and the clinical benefit observed. Participants reported the omission of this from other programmes they had taken part in, including cardiac rehabilitation. It was acknowledged that provision of support wasn't necessarily the role of the consultant, however it was important to have a trained team member who was motivated to support patients to increase and maintain their activity levels and who was knowledgeable in this area.

Discussion

Although physical activity is widely advocated to improve health, it is viewed as a significant challenge for people living with chronic health conditions and regularly increases fear. Fear of increasing physical activity was reported in the present study and as such, the need for positive reinforcement and reassurance from clinicians who are advocates of physical activity in the context of heart failure was considered necessary to overcome this barrier. Family members and partners were reported to reinforce fear, and restricted activity levels of participants for fear of an adverse event occurring. Involvement of significant others in consultations could provide a solution to this problem and should be explored further.

Participating in cardiac rehabilitation was reported to reduce psychological stress and fear associated with physical activity and was a facilitator to uptake of Active-at-Home-HF. Just like community or clinic based cardiac rehabilitation, it is important for home-based interventions to be holistic where possible[13]. Duda and colleagues reported that the success of an exercise intervention depended upon the intensity and type of support offered[14]. Active-at-Home-HF involved the use of lifestyle coaches who were trained to use specific evidence-based behaviour change techniques to target increased physical activity. They were also trained to understand the participants' condition and this was crucial for motivation/participation[15] and adherence[16].

For peer review only

The need to involve clinicians to advocate and support increased physical activity at home was considered vital to add credibility and to successfully integrate the programme in to routine clinical care. In this regard participants were more likely to participate and adhere to the programme if a clinician had advocated it and provided reassurance that it was safe. Currently, attendance at cardiac rehabilitation in hospitals or community centres frequently involves travel and associated costs, and these are barriers to participation, especially for those who have limited financial resources or who are unable to drive. Active-at-Home-HF is an alternative to help overcome these barriers while providing patients with a personalised programme capable of increasing activity levels and impacting positively on health and fitness.

A commonly suggested facilitator to ongoing engagement and maintenance of physical activity levels following completion of the programme was having an opportunity to meet with others who had completed the programme as a means of social support. This is an important consideration with 40-50% of those participating in physical activity programmes relapsing and returning to their previous physical activity states within six months[17,18].

In addition, previous studies support the use of supportive calls as an effective way to motivate patients to maintain physical activity levels, particularly those who have a low level of physical functioning[19]. Active-at-Home-HF was delivered by short telephone calls and this proved to be an effective way of targeting increased physical activity while providing support. Finally, a unanimous suggestion was a support group for heart failure patients to promote maintenance of physical activity. Such groups provide a strong form of social support where long-term friendships are developed that are essential for long-term engagement and encouragement[16]. Furthermore, these groups bring together individuals with similar conditions and work to generate a sense of obligation thereby facilitating adherence and this was highlighted by participants[20].

For peer review only

To the best of our knowledge, this is the first qualitative study to explore barriers and facilitators to participation and adherence to a home-based physical activity programme in the context of heart failure. In addition, we identified ways in which the intervention could be successfully integrated within routine clinical care. Our findings highlight that home-based physical activity programmes should be endorsed by members of the clinical team, where possible a consultant, and that clinicians should be advocates of and knowledgeable about physical activity in the context of heart failure. While it is acknowledged that not all clinical teams will have the knowledge, expertise and skills to effectively promote physical activity, the findings from our study suggest that a future intervention could incorporate a training programme to address this issue. Those who declined participation in Active-at-Home-HF did so out of fear and lack of knowledge about the benefits of physical activity in the context of heart failure. This suggests that a future intervention should incorporate specific learning/information content in addition to a recommendation from a clinical team member. Participants were supported throughout the duration of the intervention by research team members with backgrounds in exercise physiology who were trained by a Health Psychologist to use evidence-based behaviour change techniques to target increased physical activity and maintenance over time. Participants reported this support as essential to obtain feedback, maintain motivation, to challenge their perceptions and was beneficial as a form of social support. Therefore a future intervention should explore the possibility of training clinical team members to provide this support to facilitate full integration of the intervention in to routine clinical care.

Acknowledgements

The authors would like to acknowledge and thank the participants who took part in this study. This research was funded by the National Institute for Health Research Biomedical Research Centre (grant number: BH142109). LA, DJ, NCO and NO'B conceived the study and its

design. LA supervised its conduct. KB and GM facilitated recruitment of participants. SJC, SC and NCO delivered the Active-at-Home-HF intervention and conducted study visits. LA and NO'B conducted all focus group discussions; NCO, LA and NO'B analysed and interpreted data; NCO and LA drafted the manuscript. All authors revised the manuscript and approved the version to be published.

Data sharing statement

Participants were not asked to provide consent to share the transcripts of their focus group discussion beyond the research team.

References

1 O'Donovan G, Hillsdon M, Ukoumunne OC, *et al.* Objectively measured physical activity, cardiorespiratory fitness and cardiometabolic risk factors in the Health Survey for England. *Prev Med (Baltim)* 2013;**57**:201–5. doi:10.1016/j.ypmed.2013.05.022

2 Colberg SR. Physical activity: The forgotten tool for type 2 diabetes management. Front. Endocrinol. (Lausanne). 2012;**3**. doi:10.3389/fendo.2012.00070

3 Josefsson T, Lindwall M, Archer T. Physical exercise intervention in depressive disorders: Meta-analysis and systematic review. *Scand J Med Sci Sports* 2013;:1–14. doi:10.1111/sms.12050

4 British Association for Cardiovascular Prevention and Rehabilitation. National Audit of Cardiac Rehabilitation: Annual Statistical Report 2017. *Annu Rep* 2017;:1–72.https://www.bhf.org.uk/publications/statistics/national-audit-of-cardiac-rehabilitation-annual-statistical-report-2017

For peer review only

5 Piotrowicz E, Baranowski R, Bilinska M, *et al.* A new model of home-based telemonitored cardiac rehabilitation in patients with heart failure: Effectiveness, quality of life, and adherence. *Eur J Heart Fail* 2010;**12**:164–71. doi:10.1093/eurjhf/hfp181

6 Jolly K, Taylor RS, Lip GYH, *et al.* A randomized trial of the addition of home-based exercise to specialist heart failure nurse care: The Birmingham Rehabilitation Uptake Maximisation study for patients with Congestive Heart Failure (BRUM-CHF) study. *Eur J Heart Fail* 2009;**11**:205–13. doi:10.1093/eurjhf/hfn029

7 Zwisler A-D, Norton RJ, Dean SG, *et al.* Home-based cardiac rehabilitation for people with heart failure: A systematic review and meta-analysis. *Int J Cardiol* 2016;**221**:963–9. doi:10.1016/j.ijcard.2016.06.207

8 Oerkild B, Frederiksen M, Hansen JF, *et al.* Home-based cardiac rehabilitation is as effective as centre-based cardiac rehabilitation among elderly with coronary heart disease: Results from a randomised clinical trial. *Age Ageing* 2011;**40**:78–85. doi:10.1093/ageing/afq122

9 Saunders B, Sim J, Kingstone T, *et al.* Saturation in qualitative research: exploring its conceptualization and operationalization. *Qual Quant* 2018;**52**:1893–907. doi:10.1007/s11135-017-0574-8

10 Avery L, Exley C, McPherson S, *et al.* Lifestyle Behavior Change in Patients With Nonalcoholic Fatty Liver Disease: A Qualitative Study of Clinical Practice. *Clin Gastroenterol Hepatol* 2017;**15**:1968–71. doi:10.1016/j.cgh.2017.06.011

11 Tong A, Sainsbury P, Craig J. Consolidated criterio for reporting qualitative research (COREQ): a 32- item checklist for interviews and focus group. *Int J Qual Heal Care* 2007;**19**:349–57. doi:10.1093/intqhc/mzm042

For peer review only

12 Braun V, Clarke V. Braun, V ., Clarke, V .Using thematic analysis in psychology., 3:2 (2006), 77-101. *Qual Res Psychol* 2006;**3**:77–101. doi:10.1191/1478088706qp063oa

13 Haase AM, Taylor AH, Fox KR, *et al.* Rationale and development of the physical activity counselling intervention for a pragmatic TRial of Exercise and Depression in the UK (TREAD-UK). Ment. Health Phys. Act. 2010;**3**:85–91. doi:10.1016/j.mhpa.2010.09.004

14 Duda JL, Williams GC, Ntoumanis N, *et al.* Effects of a standard provision versus an autonomy supportive exercise referral programme on physical activity, quality of life and well-being indicators: a cluster randomised controlled trial. *Int J Behav Nutr Phys Act* 2014;**11**:10. doi:10.1186/1479-5868-11-10

15 O'Sullivan TL, Fortier MS, Faubert C, *et al.* Interdisciplinary physical activity counseling in primary care: a qualitative inquiry of the patient experience. *J Health Psychol* 2010;**15**:362–72. doi:10.1177/1359105309350516

16 Moore GF, Moore L, Murphy S. Facilitating adherence to physical activity: exercise professionals' experiences of the National Exercise Referral Scheme in Wales: a qualitative study. *BMC Public Health* 2011;**11**:935. doi:10.1186/1471-2458-11-935

17 Dishman RK. Increasing and maintaining physical activity and exercise. *Behav Ther* 1991;**22**:345–78. doi:10.1016/S0005-7894(05)80371-5

18 Hambrecht R, Gielen S, Linke a, *et al.* Effects of exercise training on left ventricular function and peripheral resistance in patients with chronic heart failure: A randomized trial. *JAMA* 2000;**283**:3095–101. doi:jci00033 [pii]

19 Yates BC, Anderson T, Hertzog M, *et al.* Effectiveness of follow-up booster sessions in improving physical status after cardiac rehabilitation: Health, behavioral, and

For peer review only

clinical outcomes. *Appl Nurs Res* 2005;**18**:59–62. doi:10.1016/j.apnr.2004.06.012

20 Podlog L, Dionigi R a. Psychological need fulfillment among workers in an exercise intervention: a qualitative investigation. *Res Q Exerc Sport* 2009;**80**:774–87. doi:10.1080/02701367.2009.10599619

For peer review only

COREQ (COnsolidated criteria for REporting Qualitative research) Checklist

A checklist of items that should be included in reports of qualitative research. You must report the page number in your manuscript where you consider each of the items listed in this checklist. If you have not included this information, either revise your manuscript accordingly before submitting or note N/A.

Topic	Item No.	Guide Questions/Description	Reported on Page No.
**Domain 1: Research team and reflexivity**			
Personal characteristics			
Interviewer/facilitator	1	Which author/s conducted the interview or focus group?	
Credentials	2	What were the researcher's credentials? E.g. PhD, MD	
Occupation	3	What was their occupation at the time of the study?	
Gender	4	Was the researcher male or female?	
Experience and training	5	What experience or training did the researcher have?	
Relationship with participants			
Relationship established	6	Was a relationship established prior to study commencement?	
Participant knowledge of the interviewer	7	What did the participants know about the researcher? e.g. personal goals, reasons for doing the research	
Interviewer characteristics	8	What characteristics were reported about the inter viewer/facilitator? e.g. Bias, assumptions, reasons and interests in the research topic	
**Domain 2: Study design**			
Theoretical framework			
Methodological orientation and Theory	9	What methodological orientation was stated to underpin the study? e.g. grounded theory, discourse analysis, ethnography, phenomenology, content analysis	
Participant selection			
Sampling	10	How were participants selected? e.g. purposive, convenience, consecutive, snowball	
Method of approach	11	How were participants approached? e.g. face-to-face, telephone, mail, email	
Sample size	12	How many participants were in the study?	
Non-participation	13	How many people refused to participate or dropped out? Reasons?	
Setting			
Setting of data collection	14	Where was the data collected? e.g. home, clinic, workplace	
Presence of non-participants	15	Was anyone else present besides the participants and researchers?	
Description of sample	16	What are the important characteristics of the sample? e.g. demographic data, date	
Data collection			
Interview guide	17	Were questions, prompts, guides provided by the authors? Was it pilot tested?	
Repeat interviews	18	Were repeat inter views carried out? If yes, how many?	
Audio/visual recording	19	Did the research use audio or visual recording to collect the data?	
Field notes	20	Were field notes made during and/or after the inter view or focus group?	
Duration	21	What was the duration of the inter views or focus group?	
Data saturation	22	Was data saturation discussed?	
Transcripts returned	23	Were transcripts returned to participants for comment and/or	

For peer review only

Topic	Item No.	Guide Questions/Description	Reported on Page No.
		correction?	
**Domain 3: analysis and findings**			
Data analysis			
Number of data coders	24	How many data coders coded the data?	
Description of the coding tree	25	Did authors provide a description of the coding tree?	
Derivation of themes	26	Were themes identified in advance or derived from the data?	
Software	27	What software, if applicable, was used to manage the data?	
Participant checking	28	Did participants provide feedback on the findings?	
Reporting			
Quotations presented	29	Were participant quotations presented to illustrate the themes/findings? Was each quotation identified? e.g. participant number	
Data and findings consistent	30	Was there consistency between the data presented and the findings?	
Clarity of major themes	31	Were major themes clearly presented in the findings?	
Clarity of minor themes	32	Is there a description of diverse cases or discussion of minor themes?	

Developed from: Tong A, Sainsbury P, Craig J. Consolidated criteria for reporting qualitative research (COREQ): a 32-item checklist for interviews and focus groups. *International Journal for Quality in Health Care*. 2007. Volume 19, Number 6: pp. 349 – 357

Once you have completed this checklist, please save a copy and upload it as part of your submission. DO NOT include this checklist as part of the main manuscript document. It must be uploaded as a separate file.

For peer review only

BMJ Open

Overcoming barriers to engagement and adherence to a home-based physical activity intervention for patients with heart failure: A qualitative focus group study

Journal:	*BMJ Open*
Manuscript ID	bmjopen-2019-036382.R1
Article Type:	Original research
Date Submitted by the Author:	16-Apr-2020
Complete List of Authors:	Okwose, Nduka; Newcastle University Faculty of Medical Sciences, Translational and Clinical Research Institute<br>O'Brien, Nicola; Northumbria University - City Campus, Department of Psychology<br>Charman, Sarah; Newcastle University Faculty of Medical Sciences, Translational and Clinical Research Institute<br>Cassidy, Sophie; Newcastle University Faculty of Medical Sciences, Translational and Clinical Research Institute<br>Brodie, David; Buckinghamshire New University, School of Health and Social Sciences<br>Bailey, Kristian; Newcastle upon Tyne Hospitals NHS Foundation Trust<br>MacGowan, Guy; Newcastle Upon Tyne Hospitals NHS Foundation Trust, Cardiology<br>Jakovljevic, Djordje; Newcastle University Faculty of Medical Sciences, Translational and Clinical Research Institute<br>Avery, Leah; Teesside University, School of Health & Life Sciences
**Primary Subject Heading**:	Cardiovascular medicine
Secondary Subject Heading:	Cardiovascular medicine, Qualitative research, Rehabilitation medicine, Sports and exercise medicine
Keywords:	Heart failure < CARDIOLOGY, QUALITATIVE RESEARCH, Adult cardiology < CARDIOLOGY

SCHOLARONE™
Manuscripts

I, the Submitting Author has the right to grant and does grant on behalf of all authors of the Work (as defined in the below author licence), an exclusive licence and/or a non-exclusive licence for contributions from authors who are: i) UK Crown employees; ii) where BMJ has agreed a CC-BY licence shall apply, and/or iii) in accordance with the terms applicable for US Federal Government officers or employees acting as part of their official duties; on a worldwide, perpetual, irrevocable, royalty-free basis to BMJ Publishing Group Ltd ("BMJ") its licensees and where the relevant Journal is co-owned by BMJ to the co-owners of the Journal, to publish the Work in this journal and any other BMJ products and to exploit all rights, as set out in our licence.

The Submitting Author accepts and understands that any supply made under these terms is made by BMJ to the Submitting Author unless you are acting as an employee on behalf of your employer or a postgraduate student of an affiliated institution which is paying any applicable article publishing charge ("APC") for Open Access articles. Where the Submitting Author wishes to make the Work available on an Open Access basis (and intends to pay the relevant APC), the terms of reuse of such Open Access shall be governed by a Creative Commons licence – details of these licences and which Creative Commons licence will apply to this Work are set out in our licence referred to above.

Other than as permitted in any relevant BMJ Author's Self Archiving Policies, I confirm this Work has not been accepted for publication elsewhere, is not being considered for publication elsewhere and does not duplicate material already published. I confirm all authors consent to publication of this Work and authorise the granting of this licence.

For peer review only

Overcoming barriers to engagement and adherence to a home-based physical activity intervention for patients with heart failure: A qualitative focus group study

Nduka C Okwose, PhD[1] , Nicola O'Brien, PhD[2,3], Sarah J Charman, PhD[1], Sophie Cassidy, PhD[1], David A Brodie[4] DSci, Kristian Bailey, MD[5] , Guy A MacGowan, MD[,5,6] , Djordje G Jakovljevic, PhD[1,5,] *Leah Avery, PhD[2,6]

[1] Cardiovascular Research Centre; Translational and Clinical Research Institute, Faculty of Medical Sciences, Newcastle University, UK

[2]Population Health Sciences Research Institute, Faculty of Medical Sciences, Newcastle University, UK

[3] Department of Psychology, Northumbria University, UK

[4] School of Health and Social Sciences, Buckinghamshire New University, Uxbridge, UK

[5] Newcastle upon Tyne Hospitals NHS Foundation Trust, Newcastle upon Tyne, UK

[6]Biosciences Institute, Faculty of Medical Sciences, Newcastle University, UK

[6] School of Health and Life Sciences, Teesside University, Tees Valley, UK

Corresponding Author: Dr Leah Avery: leah.avery@tees.ac.uk, Centre for Rehabilitation, Exercise and Sports Sciences, School of Health and Life Sciences, Teesside University, Tees Valley, UK. TS1 3BA. Tel: +44 (0)1642 384130; Email: leah.avery@tees.ac.uk.

For peer review only

Abstract

Objectives: Clinical guidelines recommend regular physical activity for patients with heart failure to improve functional capacity and symptoms and to reduce hospitalisation. Cardiac rehabilitation programmes have demonstrated success in this regard, however uptake and adherence are sub-optimal. Home-based physical activity programmes have gained popularity to address these issues, although it is acknowledged that their ability to provide personalised support will impact upon their effectiveness. The study aimed to identify barriers and facilitators to engagement and adherence to a home-based physical activity programme, and to identify ways in which it could be integrated into the care pathway for patients with heart failure.

Design: A qualitative focus group study was conducted. Data were analysed using thematic analysis.

Participants: A purposive sample of 16 patients, 82% males, aged 68 ± 7 years, with heart failure duration 10 ±9 years were recruited.

Intervention: A 12 week behavioural intervention targeting physical activity was delivered once per week by telephone.

Results: Ten main themes were generated that provided a comprehensive overview of the active ingredients of the intervention in terms of engagement and adherence. Fear of undertaking physical activity was reported to be a significant barrier to engagement. Influences of family members were both barriers and facilitators to engagement and adherence. Facilitators included endorsement of the intervention by clinicians knowledgeable about physical activity in the context of heart failure; ongoing support and personalised feedback from team members, including tailoring to meet individual needs, overcome barriers and increase confidence.

For peer review only

Conclusions: Endorsement of interventions by clinicians to reduce patients' fear of undertaking physical activity and individual tailoring to overcome barriers are necessary for long-term adherence. Encouraging family members to attend consultations to address misconceptions and fear about the contraindications of physical activity in the context of heart failure should be considered for adherence, and peer-support long-term maintenance.

Strengths and limitations of this study

- Qualitative focus groups are an appropriate methodological approach to explore views on uptake, engagement and adherence to an intervention and can help facilitate consensus
- Adults with heart failure who had declined participation in the intervention and those who had previously attended and declined participation in centre-based programmes were recruited to obtain representativeness of views
- A higher proportion of men participated, therefore the views of women should be explored further
- The outcome of the methodological approach undertaken will inform intervention optimisation to support delivery during routine clinical care

Introduction

Increasing levels of physical activity is recommended to improve cardio-metabolic outcomes and to enhance overall health and psychological wellbeing[1–3]. In the UK, cardiac rehabilitation is offered as a pathway of referral for people living with chronic conditions such as cardiovascular disease. Encouraging patients to attend is often challenging, although uptake

For peer review only

of cardiac rehabilitation by patients following a cardiac event or procedure (e.g., MI, PCI) increased from 45% in 2013 to 50% in 2016[4]. However, these figures provide an artificial estimate of attendance in the context of heart failure, because only 5.3% of patients with heart failure actually attend cardiac rehabilitation[4]. Furthermore, dropout rates often exceed 20% for those attending centre-based programmes[5]. Barriers to uptake, engagement and adherence are reported to include proximity of cardiac rehabilitation services and motivation levels of patients to attend[5,6].

A potential solution to overcome these barriers is the provision of home-based physical activity programmes. These have shown to elicit similar outcomes to centre-based programmes and could be particularly attractive to those with a preference to increase activity levels at home. They also have the potential to be tailored to individual needs and preferences, thereby increasing engagement, adherence and reducing dropout rates[7,8].

This qualitative study explored the barriers and facilitators to engagement and adherence to a home-based physical activity intervention for patients with heart failure, called 'Active-at-Home-HF'. The findings will be used to inform optimisation of the intervention for delivery by clinicians during routine care delivery.

For peer review only

Methods

This study was approved by the NHS North East – Tyne and Wear South Research Ethics Committee (REC reference: 15/NE/0190). All participants provided informed written consent prior to participation.

Intervention

Active-at-Home-HF is a behavioural intervention designed to support patients with heart failure to increase their baseline physical activity levels by at least 2000 steps per day over a period of 12 weeks **[9].** The intervention was developed as an alternative to centre-based cardiac rehabilitation programmes to help overcome some of the well-documented barriers to engagement and adherence (e.g., travel, cost, group-based), but also as a means to support those who have previously attended centre-based programmes who wish to continue physical activity at home. Three researchers with backgrounds in exercise physiology were trained by a Chartered Health Psychologist to deliver the intervention adopting the role of a lifestyle coach. Coaches used evidence-based behaviour change techniques including behavioural goal setting, action planning, problem solving, self-monitoring, feedback on behaviour and review of behavioural goals [10, 11, 12]. Participants were supported to increase and sustain physical activity levels via telephone calls lasting between 8 and 15 minutes on at least 10 occasions, i.e. weekly over a period of 12 weeks. The number of telephone calls was dictated by the participant up to a maximum of 12 calls (i.e. one call per week). Physical activity goals were set and modified in collaboration with the coach and participant depending upon each participant's progress, capability, needs and preferences, with an emphasis on duration of physical activity rather than intensity. A standardised proforma was used to structure calls and to serve as a prompt to use all behaviour change techniques, where appropriate and to increase fidelity of delivery. All three researchers who delivered the intervention also completed study visits with partipants during the Active-at-Home-HF pilot study[13].

Design

A qualitative study embedded within a pilot study of the Active-at-Home-HF intervention was conducted [13]. The aim was to deliver the intervention to participants and obtain feedback on specific aspects of it (e.g., mode of delivery), but primarily to obtain views on whether an

For peer review only

intervention of this kind could provide the level and type of support required to increase and maintain physical activity levels. Focus group discussions were used to identify barriers and facilitators to engagement and adherence, and ways in which the intervention could be integrated into routine clinical care.

Participants

Eligible participants had left ventricular ejection fraction ≤ 40% diagnosed at least three months previously; were clinically stable and receiving an optimal medical treatment; had no contraindications to physical activity; and were able to walk and perform activities of daily living independently. Table 1 presents a summary of participant characteristics.

Table 1. Mean and SD (±) patients' demographic and clinical characteristics

Parameter	
Age (years)	67±5
Men/women	13/3
Weight (kg)	85.2±15.9
Height (m)	1.73±0.1
Duration of heart failure (years)	10±9
Medication	
ACE inhibitors	12
B- blockers	16
ARBs	3
Diuretics	9
Warfarin	5
ICD/Pacemakers	10
Comorbidities	
COPD	1
Type 2 Diabetes	5
Hypertension	16
Depression	2
Arthritis	1

For peer review only

ACE, angiotensin converting enzyme; ARB, angiotensin receptor blockers; NSAID, non-steroid anti-inflammatory drugs; COPD, chronic obstructive pulmonary disease; ICD, Implantable cardioverter defibrillator

The aim was to recruit a purposive sample of between 12 and 20 participants in line with published guidance and previously published qualitative research to ensure data saturation[13,14]. Eligible participants included those who had previously taken part and those who had refused participation in centre-based cardiac rehabilitation programme to ensure a representation of views and experiences, and also included those who had refused participation in Active-at-Home-HF to identify barriers to uptake. Participants were contacted via email, telephone, or spoken to in person to discuss participation in the study and given an opportunity to ask questions to ensure they understood the aims of the study.

Study Procedure

Eligible participants under the care of a cardiologist at the Freeman Hospital, Newcastle upon Tyne, UK were invited to take part in the study. Focus group discussions were facilitated by two female Chartered Health Psychologists with expertise in health behaviour change, physical activity intervention and qualitative research methods. Neither of the researchers had met the participants prior to the focus group discussions and neither were aware of any preliminary findings of the Active-at-Home-HF pilot study. The Active-at-Home-HF behavioural intervention was developed by one of the Health Psychologists conducting the focus groups. The focus group discussions were conducted in a meeting room at the Royal Victoria Infirmary, Newcastle upon Tyne. Only the two researchers and the participants were present during the discussions. One of the researchers explained the aims and objectives of the focus group discussions to set the scene, and the role of both psychologists within the research team. This included how the data generated from the focus group discussions would be used, i.e. their goal

For peer review only

was to use the findings to further develop the intervention and/or integrate it within usual clinical care to help other people with heart failure.

Materials

The research team developed a topic guide to structure discussions. Topics included reasons for taking part, or declining participation; preference for completing a home-based programme; information required to inform decision-making regarding participation; support requirements to adhere to and complete the programme and potential barriers; and expectations of the programme. All questions were open-ended and prompts were used to generate a deeper understanding of participants' views. Each focus group discussion was audio recorded and transcribed verbatim.

Methodological Quality and reporting

The research was conducted in accordance with the consolidated criteria for reporting qualitative research (COREQ) to enhance transparency and maximise methodological quality[15]. To reduce bias from responders, members of the team delivering the intervention or involved in the pilot study clinic visits were not involved with focus group discussions.

Data Analysis

Data were analysed by hand using thematic analysis[16]. To maximise methodological quality and trustworthiness of the findings, the following analysis procedure was undertaken: (i) Two researchers independently read and re-read focus group transcripts (NCO and LA); (ii) both researchers independently applied codes to segments of data within the first focus group transcript to develop initial themes and sub-themes; (iii) the same two researchers discussed findings and agreed a preliminary set of themes and sub-themes; (iv) one researcher (NCO) repeated this process with the remaining two transcripts; and (v) both researchers agreed the final set of themes and sub-themes that adequately represented the data set following

For peer review only

discussion. Supporting direct quotes from participants were subsequently applied to themes and sub-themes. A third researcher (NO'B) reviewed themes and sub-themes generated to verify interpretation. We did not consider it necessary to use qualitative data analysis software due to the small number of focus group transcripts. The aim was to avoid deterministic processes, which can be a risk with qualitative software, and to prioritise gaining an in depth understanding of the data generated. We achieved this with in depth scrutiny of the transcripts by hand.

Patient and public involvement

There was no patient and public involvement in the design or planning of the study.

Results

Three focus groups were conducted, with a duration of 47, 53 and 83 minutes respectively. A total of sixteen individuals (13 males, 3 females; median age 68 years; interquartile range: 6.5 years) from the twenty who were eligible to take part in the pilot study, and two who were eligible but declined participation took part in a focus group discussion. Participants had either completed Active-at-Home-HF (4 participants), were still participating at the time of the focus group (10 participants), or had declined participation (2 participants). Three participants had previously declined centre-based cardiac rehabilitation. Ten participants had implanted cardiac defibrillators (ICDs). Fifteen of the sixteen participants were retired from active work. Following thematic analyses, 10 main themes were generated. It was noted following the conduct of focus group three that no new data were generated, i.e. it was agreed that data saturation was reached. Table 2 provides a summary of the themes and subthemes, and example direct quotes to provide context.

For peer review only

Table 2: A summary of themes and subthemes derived from thematic analyses of focus group discussion transcripts

Theme	Sub-theme	Quotes
**1. Fear of undertaking physical activity**		'I'm not confident when I go out on my own. I'm frightened I'm going to collapse' (FG 1, Male, aged 57)<br>'It's frightening when you've gone too far. Something's always holding me back from going that little bit further, you know' (FG 1, Male, aged 56)
**2. Family members influence physical activity efforts**	**2.1 Fear in family members prevents engaging with physical activity**	'I've got a wife who's not happy if I say I'm going out for a walk for an hour.., because she thinks that's too much' (FG 2, Male, aged 67)<br>'..Sometimes, your family don't want you to do it, not because they're being horrible, it's because they're concerned about you' (FG 1, Male, aged 78)
	**2.2 Support from family members facilitates physical activity**	'I needed motivation which I got from the family, which is great…I don't know how I would have fared without family' (FG 2, Female, aged 62)<br>'I think it does help if you've got a supportive partner. My husband has come out with me for all my walks right from the start. I think it's good if your partners can be involved and then they realise, don't they, how they can help' (FG 2, Female, aged 62)
**3. Physical activity programmes require endorsement by clinicians**		'I think my biggest breakthrough was . . . I was doing more and more but, again, family was saying, "Take it easy." Fortunately, my wife was with me on one occasion when I saw Dr "XX" and he asked what I'd been doing, and he said, "Well, do more." I said,

For peer review only

	“How do you mean by that?” He said, “Just do more and more until you feel you can’t and then back off a bit.” So, fortunately, my wife was there and she heard him say it. So, I’ve had no peace since then’ (FG 1, Male, aged 78)<br><br>‘My nurse certainly was interested [in my physical activity habits] and when I went last time, was aware of what I was doing and obviously encouraging’ (FG 2, Female, aged 62)
**4. Completion of a cardiac rehabilitation programme provides confidence to complete a physical activity programme at home**	‘I think the most important thing when you've had a heart attack is to have the confidence to do it at all. I think the cardiac rehabilitation that I had gave me the confidence and then you can move on to do other things. I think if you had just had a heart attack and someone says, "Right, you've got to go for a walk every day," it would be difficult to do. I think it would. So it's like a step in the process’ (FG 2, Male, aged 71)<br><br>‘I was really upset when it [cardiac rehabilitation] finished. I had somewhere to go. I’m sat at home by myself all day, so coming here on the Wednesday [for the study visit] and receiving feedback, it was like Christmas all over again, you know. It was great. (FG 1, Male, aged 56)
**5. Coach support increases motivation long-term**	‘[having your coach call you] encourages you to keep up with it, definitely, because I think if you didn't get the phone calls, I think you might just go, "It's not very nice out today, I'll not bother going’(FG 2, Female, aged 62)

For peer review only

	'When somebody is monitoring you, it makes you get up and go out, doesn't it? I mean if you go to the gym yourself, some mornings you might say, "I'm not going to go today," but it's helping you, isn't it?' (FG 3, Male, aged 62)
**6. Weekly agreed targets increased confidence and motivation**	'It's a target as everyone's saying and you want to do it and you feel very enthusiastic about doing it and I will certainly continue after the 12 weeks because it would be pointless stopping all together wouldn't it? It would waste the benefit sort of thing' (FG 2, Male, aged 70)<br><br>'I think if you were told increase to 12,000 and I come back in 12 weeks' time and we talk about it, you would go... but what's good is on Tuesday [my coach] rings me and goes through all the information, we get an average and then he'll ask me how I'm feeling, etc., etc., and say, "Right, let's try and take that 12,000 up to 12,500." That's on a weekly basis. You know for a fact that someone's interested in what's been happening for the past week and we can take that from where we are now to try and improve things.' (FG 2, Male, aged 69)
**7. A credible team increased the likelihood of participation**	'I think the fact that people like yourselves who are specialists in this sort of area take such an interest in us people. I think that gives you the boost again. I think it does boost people when you've got people who are really even higher than your GP and what not in that specific area of cardiac problems' (FG 2, Male, aged 78)

For peer review only

	'Well I think this is a speciality subject, what you're doing and all the stuff here that we've been doing. I think it's better in that situation to get the right advice' (FG 2, Male, aged 67)
**8. The surrounding environment creates barriers to increased physical activity/exercise**	'I think another …. difficulty… trying to get people motivated is the area that they live in. Now if you've got a bit of countryside, open fields and that, it opens you to more space unlike just got solid concrete, trying to get people motivated to walk down the same street or go a particular… way, that's going to be difficult I think. If you're going to motivate people you're going to have to think where they actually live because people need different types of motivation. For people who live near the coast and the countryside, people living smack bang in the middle of a built-up area etc' (FG 2, Male, aged 67)<br>'To me, I'm okay most of the time on the flat, but it's any incline. The littlest incline in the world kills me' (FG 2, Male, aged 78)<br>'I think some of the benefit was the climate as well, it's a struggle in wind and rain' (FG 3, Female, aged 63)
**9. Participation prompts an increase in every day activity levels**	'What I do now is I have to drive to the supermarket but I park in the furthest corner of the car park and I walk round the car park. Then on rainy days, what I have been doing is going into the supermarket and going round it twice before I start my shopping. The people must think you're mad if they look at you on the CCTV' (FG 2, Male, aged 71)

For peer review only

		‘I think it's got to work doing exercise. I mean they brought me in here; they're going on about putting a defibrillator in. Now when he saw me, how my heart had changed just through doing things, he decided he wouldn't put one in’ (FG 3, Male, aged 62)<br><br>‘I haven't been back to see my specialist at the Freeman [hospital], because he doesn't want to see me for 12 months because I'm too fit for him. I said, "Are you sick of me?" he said, "No." It was six months before and then I went for the last check sometime last year, he said, "Right, I don't want to see you for 12 months. I said, have you gone off me?’ (FG 2, Male, aged 71)<br><br>‘Well since I've completed the course [Active-at-Home-HF], I've been diabetic for 30 years, my blood sugar levels have never been as normal as an ordinary person's in my life’ (FG 2, Male, aged 79)
**10. Support to maintain long-term activity levels would be beneficial**	**10.1 Support from a healthcare professional following completion of the programme would help maintain increased physical activity**	‘I think a follow-up is a good idea. You need some sort of follow-up after you've finished. How they do it, whether it's a phone call or a meeting with your doctor or whatever, your GP or anybody like that, I don't know how they would do it. But I think that's quite important that. Then you wouldn't feel as if you've been chucked on the scrap heap type of thing, you've finished, it's done’ (FG 3, Male, aged 73)<br><br>‘Do you know, that’s the worst thing, when you’ve finished your course and the following Wednesday there’s no phone call. It’s horrendous isn’t it?’ (FG 2, Male, aged 67)

For peer review only

**10.2 Group peer support would promote long-term physical activity**	'I know it's just the start of this programme …, As far as feedback on it is concerned, maybe something about halfway through, maybe six weeks you could have a little meeting with some of the people just for half an hour, just have a little chat and see who's there, what's what, talk to people, something like that anyway. A little informal meeting or a social evening or whatever you want to call it. It lets everybody else know that it's not just you or another two or three people, it might be 20 people' (FG 3, Male, aged 73)

For peer review only

Fear of undertaking physical activity

Once diagnosed with heart failure, participants reported difficulties overcoming the fear associated with the diagnosis (i.e. future cardiac events and overexertion) and increasing physical activity exacerbated this fear. While some reported that they could still engage in some physical activity, there was concern that too much would be detrimental. This was reported to be a significant barrier to participation: *'It's frightening when you've gone too far. Something's always holding me back from going that little bit further, you know'* (FG 1, Male, aged 56). In this regard, it was reported that reassurance from a clinican is required to confirm that increasing physical activity is safe, and specific information in terms of how much activity should be undertaken to be beneficial, and not detrimental.

Family members influence engagement with physical activity

It was reported that family members were both a barrier and facilitator to engaging in physical activity. Some participants reported that family members dissuaded them from engaging in physical activity. Although, there was a consensus that this was linked to fear and concern *'Sometimes, your family don't want you to do it, not because they're being horrible, it's because they're concerned about you'* (FG 2, Male, aged 78). Fear of family members often meant that participants were less active following a diagnosis of heart failure than they were previously.

It was also reported that family members were a strong motivating factor once they were reassured that increasing physical activity was beneficial. For some participants, having a partner or relative to support them and participate in new activity routines was reported as a significant facilitator to continued participation. A typical quote included: *'I think it does help if you've got a supportive partner. My husband has come out with me for all my walks right*

For peer review only

from the start. I think it's good if your partners can be involved and then they realise, don't they, how they can help' (FG 2, Female, aged 62). There was a consensus that without the practical and emotional support of partners and relatives, increasing physical activity would be more difficult.

Physical activity programmes require endorsement by clinicians

Endorsement of the physical activity programme by a clinician was reported to reduce patients' fears about increasing levels of physical activity and potentially making their condition worse. Furthermore, it reassured family members when they witnessed clinicians explaining the benefits and advising an increase. In addition, ongoing positive reinforcement by the clinical team was considered paramount to continued participation: '*My nurse certainly was interested [in my physical activity levels] and when I went last time, was aware of what I was doing and was encouraging'* (FG 2, Female, aged 62).

Prior completion of a cardiac rehabilitation programme provides confidence to engage with home-based physical activity programmes

Cardiac rehabilitation was reported to play a significant role in confidence building among heart failure patients, including confidence to continue with activities of daily living. As such it was reported to be a significant facilitator to uptake of Active-At-Home-HF as a means of providing patients with ongoing support and encouragement to be physically active in the long-term. Continued participation and feedback was particularly important to those who had completed a cardiac rehabilitation programme: *'I was really upset when it [cardiac rehabilitation] finished. I had somewhere to go. I'm sat at home by myself all day, so coming*

For peer review only

here on the Wednesday [for the study visit] and receive feedback, it was like Christmas all over again, you know. It was great' (FG 1, Male, aged 56).

Behavioural support provided by a trained professional increases motivation and adherence

Participants reported that having a trained professional who monitored their performance and provided personalised advice and support promoted continued participation (i.e. adherence): *'[having your coach call you] encourages you to keep up with it, definitely, because I think if you didn't get the phone calls, I think you might just go, "It's not very nice out today, I'll not bother going'*(FG 2, Female, aged 62). It was important that the support was provided by someone who could advise on the type, amount and duration of activity required to reach a specific target; and someone who was skilled in the use of behavioural strategies who could appropriately challenge participants and support them to overcome perceived barriers.

Agreed weekly targets increased confidence and motivation

Physical activity goal setting was considered an important mechanism to increase motivation, particularly when weekly goals were personalised and agreed between the participant and coach. This provided reassurance that goals were realistic, attainable and safe and it was reported to be rewarding when goals were achieved. Some participants were keen to set themselves daily or weekly targets once they had knowledge and confidence in their capabilities, highlighting the potential long-term impact of the programme.

For peer review only

> *'It's a target as everyone's saying and you want to do it and you feel very enthusiastic about doing it and I will certainly continue after the 12 weeks because it would be pointless stopping all together wouldn't it?'* (FG 2, male, aged 70).

A credible team increases the likelihood of participation

An incentive of taking part in Active-at-Home-HF was reported to be the support and monitoring offered by experts in heart failure and physical activity. Participants emphasised that endorsement of the programme by a Consultant who was knowledgeable about the benefits of physical activity in the context of heart failure, and a referral to a trained professional/member of his or her team who was aware of their clinical circumstances helped overcome barriers to ongoing participation. The 'knowledgeable team' provided reassurance around safety, and was skilled in tailoring the intervention to individual needs and preferences and this was considered important to facilitate initial engagement and adherence.

> *'Well I think this is a speciality subject, what you're doing and all the staff here know what we've been doing. I think it's better in that situation to get the right advice'* (FG 2, Male, aged 67).

The surrounding environment creates barriers to increasing physical activity and maintaining a physically active lifestyle

Environmental factors including living in hilly areas or in surroundings that were not considered picturesque were reported to be barriers to engagement and ongoing participation in physical activity. Poor weather conditions were most frequently reported to impact negatively on motivation and long-term participation.

For peer review only

'To me, I'm okay most of the time on the flat, but it's any incline. The littlest incline in the world kills me' (FG 2, Male, aged 78).

'I think some of the benefit was the climate as well, because it was through the summer' (FG 3, Female, aged 63).

Participants reported that the support they received via Active-at-Home-HF enabled them to identify ways in which they could increase their levels of activity despite these barriers, for example planning alternative ways to increase their step count and reach their overall goals. In this regard the programme increased motivation and realisation that there was an alternative approach to increasing activity to a level sufficient to have a clinically meaningful impact.

For peer review only

Participation prompts an increase in every day activity levels and leads to unexpected positive outcomes

Active-at-Home-HF was reported to encourage participants to seek ways to increase their activity levels by modifying their everyday behaviours (e.g. walking instead of driving). One participant reported how the intervention prompted him to spend more time with his grandchildren, thereby positively affecting activity levels and social connections with his family. Some described how their fitness and feeling of wellbeing had improved (this was confirmed in the pilot study) and the frequency of appointments with their consultant had reduced following progress.

'I haven't been back to see my specialist at the hospital, because he doesn't want to see me for 12 months because I'm too fit for him. I said, "Are you sick of me?" he said, "No." It was six months before and then I went for the last check sometime last year, he said, "Right, I don't want to see you for 12 months' (FG 2, male, aged 71).

The intervention led to improvements in cardiac function for the majority of those who participated[9]. For some, this outcome meant avoiding invasive procedures that were planned prior to the study. For other participants the intervention helped to improve cardiac function and control other co-morbidities[9].

> *'Well since I've completed the course [Active-at-Home-HF], I've been diabetic for 30 years, my blood sugar levels have never been as normal as an ordinary person's in my life'* (FG 2, male, aged 79).

Ongoing support would facilitate long-term maintenance of physical activity

Continued support was considered critically important for psychological and emotional wellbeing as well as programme adherence and maintenance of activity levels following completion of the intervention. It was emphasised that support should include a review of progress to promote ongoing motivation.

> *'Do you know, that's the worst thing, when you've finished your course and the following Wednesday there's no phone call. It's horrendous isn't it?'* (FG 2, male, aged 67).

There was a general consensus that having meetings with others who have taken part in the programme would be beneficial for social support and promoting increased physical activity levels long-term.

> *'Maybe something about halfway through, maybe six weeks you could have a little meeting with some of the people just for half an hour, just have a little chat and see who's there, what's what, talk to people, something like that anyway. A little informal meeting or a social evening or whatever you want to call it. It lets everybody else know*

For peer review only

that it's not just you or another two or three people, it might be 20 people' (FG 3, male, aged 73).

A consistent theme across focus group discussions was the need for ongoing support to maintain physical activity levels and the clinical benefit observed. Participants reported the omission of this from other programmes they had taken part in, including cardiac rehabilitation. It was acknowledged that provision of support wasn't necessarily the role of the consultant, however it was important to have a trained team member who was motivated to support patients to increase and maintain their activity levels and who was knowledgeable in this area. Peer-support was considered important for long-term maintenance of physical activity following completion of the programme as a means of continuing activity consistently and safely.

Discussion

Although physical activity is widely advocated to improve health, it is viewed as a significant challenge for people living with chronic health conditions and regularly increases fear. Fear of increasing physical activity was reported in the present study and as such, the need for positive reinforcement and reassurance from clinicians who are advocates of physical activity in the context of heart failure was considered necessary to overcome this barrier. Family members and partners were reported to reinforce fear, and restricted activity levels of participants for fear of an adverse event occurring. Involvement of significant others in consultations could provide a solution to this problem and should be explored further.

Participating in cardiac rehabilitation was reported to reduce psychological stress and fear associated with physical activity and was a facilitator to uptake of Active-at-Home-HF. Just like community or clinic based cardiac rehabilitation, it is important for home-based interventions to be holistic where possible[17]. Duda and colleagues reported that the success of an exercise intervention depended upon the intensity and type of support offered[18].

For peer review only

Active-at-Home-HF involved the use of lifestyle coaches who were trained to use specific evidence-based behaviour change techniques to target increased physical activity, and this training was essential to provide the necessary skills and confidence to effectively deliver the intervention. Although coaches, through their academic training, were able to communicate the physiological and health benefits of increased physical activity in the context of heart failure, which is important for motivation/engagement[19] and adherence[20], they reported that without behavioural training they would have struggled specifically to support participants to overcome barriers and this would have impacted upon adherence. This suggests that training of those required to deliver the intervention is critically important to support enactment and maintenance of physical activity. Therefore, behavioural training and the provision of a standardised proforma were considered facilitators to intervention delivery.

The need to involve clinicians to advocate and support increased physical activity at home was considered vital to reassure patients about safety and efficacy, to add credibility and to successfully integrate the programme in to routine clinical care. In this regard participants were more likely to participate and adhere to the programme if a clinician had advocated it and were made aware of their progress. A potential limitation of our study is that only two individuals who declined participation in Active-at-Home-HF took part in a focus group discussion. This provides limited insights in terms of barriers to uptake and this should be explored further. Although, encouragingly, 25% of our sample had previously declined participation in centre-based cardiac rehabilitation programmes. Our findings suggest that the decision to participate in Active-at-Home-HF was largely due to the way in which it was offered to patients by clinicians who were positive about the programme, and who could provide reassurance about its safety and overall benefits.

Currently, attendance at cardiac rehabilitation in hospitals or community centres involves frequent travel and associated costs, and these are barriers to participation, especially for those

For peer review only

who have limited financial resources or who are unable to drive. Furthermore, for some patients a group-based environment reduces confidence and doesn't always provide the one-to-one support they require. Active-at-Home-HF is an alternative to help overcome these barriers while providing patients with a personalised programme capable of increasing activity levels and impacting positively on health and fitness.

Previous studies support the use of supportive calls as an effective way to motivate patients to maintain physical activity levels, particularly those who have a low level of physical functioning[21]. The Active-at-Home-HF intervention was delivered by short telephone calls and this proved to be an effective way of targeting and maintaining increased physical activity while providing support. The brief nature of these telephone interactions increases the likelihood that this intervention could be feasibly rolled out within routine clinical practice. However, our training programme focussed only on the delivery of behavioural strategies by coaches who were already knowledgeable about the role of physical activity in the context of heart failure. Therefore, this suggests the potential need for training developed specifically for clinicians to ensure they have the knowledge and skills required to effectively deliver all aspects of the intervention. A commonly suggested facilitator to ongoing engagement and maintenance of physical activity levels following completion of the programme was having an opportunity to meet with others who had completed the programme as a means of social support. This is an important consideration with 40-50% of those participating in physical activity programmes relapsing and returning to their previous physical activity states within six months[21,22]. Such groups provide a strong form of social support where long-term friendships are developed that are essential for long-term engagement and encouragement[20]. Furthermore, these groups bring together individuals with similar conditions and work to generate a sense of obligation thereby facilitating adherence and this was highlighted by participants[23]. It is important to highlight that group support was suggested mainly at the

For peer review only

post intervention time-point to promote maintenance. This reinforces the need for some to gain mastery and confidence around increasing their activity levels before entering a group environment.

To the best of our knowledge, this is the first qualitative study to explore barriers and facilitators to participation and adherence to a home-based physical activity programme in the context of heart failure. In addition, we identified ways in which the intervention could be successfully integrated within routine clinical care. Our findings highlight that facilitators to home-based physical activity programmes include endorsement by members of the clinical team, where possible a consultant, and that clinicians should be advocates of and knowledgeable about physical activity in the context of heart failure. While it is acknowledged that not all clinical teams will have the knowledge, expertise and skills to effectively promote physical activity, the findings from our study suggest that a future intervention should incorporate training for clinicians to address this issue, and this would help facilitate future roll-out. Those who declined participation in Active-at-Home-HF did so out of fear and lack of knowledge about the specific benefits of physical activity in the context of heart failure. Specifically, participants believed that physical activity wouldn't improve their condition and fear of a cardiac event outweighed any possible benefits of an intervention. Findings also suggested that partners/family members reinforced fear and beliefs about efficacy. This suggests that a future intervention should incorporate specific learning/education content about the role of physical activity in addition to a recommendation from a clinical team member, and involve family members, where possible to overcome misconceptions. Participants were supported throughout the duration of the intervention by research team members with backgrounds in clinical exercise physiology who were trained by a Health Psychologist to use evidence-based behaviour change techniques to target increased physical activity and maintenance over time. Participants reported this support as essential to obtain feedback,

For peer review only

maintain motivation, to challenge their perceptions and it was beneficial as a form of social support. Therefore a future intervention should involve training of clinical team members to provide this support to facilitate full integration of the intervention in routine clinical care.

In summary, the most salient facilitators to uptake and engagement of the Active-at-Home-HF intervention were: clinical team members who provided personalised information about the benefits and safety of the intervention, and involvement of family members during consultations to provide them with reassurance and encourage them to provide support. In the context of health behaviour change theory, this approach targets attitudes, beliefs, risk perceptions and self-efficacy of patients that increases the likelihood of intention formation (i.e. engagement with the programme). Facilitators to adherence included behavioural prompting (i.e. a telephone call) by a knowledgeable team member who can provide individualised feedback and support to enable patients to set and revise activity goals, and identify ways to overcome barriers to reaching these goals. In combination these self-regulatory strategies target adherence and support maintenance of physical activity. Positive reinforcement from a consultant (e.g., positive feedback on increased activity levels in relation to outcomes) was considered a further facilitator to adherence. Although involvement of family members wasn't a formal component of the Active-at-Home intervention, in instances where partipants did involve family members in their consultations they reported this to be of significant benefit to adherence. Future delivery of an optimised version of this intervention should consider uptake, engagement and adherence separately and use the facilitators identified to target each.

Although it's possible that patients referred to rehabilitation with other long-term health conditions can present with unique challenges that create barriers to uptake, engagement and adherence, the findings of our qualitative study revealed parallels with other clinical groups. A 2018 systematic review of qualitative studies in the context of COPD reported fear and lack of

For peer review only

self-efficacy as barriers to uptake and engagement, and the relationship with healthcare professionals, personalised feedback, and peer interaction as facilitators to engagement and adherence[24]. Similarly in the context of stroke, barriers and facilitators included fear of a subsequent stroke, and personalised feedback and support to promote adherence[25]. These findings suggest that commonalities exist, and the way in which we communicate and support patients to increase and maintain physical activity levels is vitally important. As such there is potential to adapt the Active-at-Home-HF intervention for delivery in a range of contexts.

Funding

This research was funded by the National Institute for Health Research Biomedical Research Centre (grant number: BH142109). The funder and study sponsor played no role in the study design; collection, analysis and interpretation of data; writing the manuscript; or the decision to submit the manuscript for publication.

Competing Interest

The authors declare that they have no competing interests

Author Contributions

LA, DJ, and NCO conceived the study and its design. LA supervised its conduct. KB and GM facilitated recruitment of participants and collected demographic data. SJC, SC and NCO delivered the Active-at-Home-HF intervention and conducted study visits that involved data collection. LA and NO'B conducted all focus group discussions; NCO, LA and NO'B analysed and interpreted data; NCO and LA drafted the manuscript. All authors LA, NCO, DJ, DB, KB, GM, SJC, SC and NO'B made an important intellectual contribution, revised the manuscript and approved the version to be published. LA is accountable for all aspects of the study, including the accuracy and integrity of the data.

For peer review only

Acknowledgements

The authors would like to acknowledge and thank the participants who took part in this study.

Data sharing statement

Participants were not asked to provide consent to share the transcripts of their focus group discussion beyond the research team.

For peer review only

References

1 O'Donovan G, Hillsdon M, Ukoumunne OC, et al. Objectively measured physical activity, cardiorespiratory fitness and cardiometabolic risk factors in the Health Survey for England. *Prev Med* 2013;57:201–5.

2 Colberg SR. Physical activity: The forgotten tool for type 2 diabetes management. *Front Endocrinol* 2012;3:70.

3 Josefsson T, Lindwall M, Archer T. Physical exercise intervention in depressive disorders: Meta-analysis and systematic review. *Scand J Med Sci Sports* 2014; 24:259-72.

4 British Association for Cardiovascular Prevention and Rehabilitation. National Audit of Cardiac Rehabilitation: Annual Statistical Report 2017. *Annu Rep* 2017;:1–72.https://www.bhf.org.uk/publications/statistics/national-audit-of-cardiac-rehabilitation-annual-statistical-report-2017

5 Piotrowicz E, Baranowski R, Bilinska M, et al. A new model of home-based telemonitored cardiac rehabilitation in patients with heart failure: Effectiveness, quality of life, and adherence. *Eur J Heart Fail* 2010;12:164–71.

6 Jolly K, Taylor RS, Lip GYH, et al. A randomized trial of the addition of home-based exercise to specialist heart failure nurse care: The Birmingham Rehabilitation Uptake Maximisation study for patients with Congestive Heart Failure (BRUM-CHF) study. *Eur J Heart Fail* 2009;11:205–13.

7 Zwisler A-D, Norton RJ, Dean SG, et al. Home-based cardiac rehabilitation for people with heart failure: A systematic review and meta-analysis. *Int J Cardiol* 2016;221:963–9.

8 Oerkild B, Frederiksen M, Hansen JF, *et al.* Home-based cardiac rehabilitation is as effective as centre-based cardiac rehabilitation among elderly with coronary heart disease: Results from a randomised clinical trial. *Age Ageing* 2011;40:78–85.

9 Okwose N, Avery L, O'Brien N, et al. Acceptability, Feasibility and Preliminary Evaluation of a Novel, Personalised, Home based Physical Activity Intervention for Chronic Heart Failure (Active-at-Home-HF): A Pilot Study. *Sports Med Open*, 2019; 5: 45.

10 Avery L, Flynn D, Van Wersch A, et al. Changing physical activity behavior in type 2 diabetes: A systematic review and meta-analysis of behavioral interventions. *Diabetes Care*. 2012;35:2681–9.

11 Avery L, Flynn D, Dombrowski SU, et al. Successful behavioural strategies to increase physical activity and improve glucose control in adults with Type 2 diabetes. *Diabet Med*. 2015;32:1058–62

12 Avery L, Charman SJ, Taylor L et al. Systematic development of a theory-informed multifaceted behavioural intervention to increase physical activity of adults with Type 2 diabetes in routine primary care: Movement as Medicine for Type 2 Diabetes, Imp Sci,11,99.

For peer review only

13 Saunders B, Sim J, Kingstone T, et al. Saturation in qualitative research: exploring its conceptualization and operationalization. *Qual Quant* 2018;52:1893–907.

14 Avery L, Exley C, McPherson S, et al. Lifestyle Behavior Change in Patients With Nonalcoholic Fatty Liver Disease: A Qualitative Study of Clinical Practice. *Clin Gastroenterol Hepatol* 2017;15:1968–71.

15 Tong A, Sainsbury P, Craig J. Consolidated criteria for reporting qualitative research (COREQ): a 32- item checklist for interviews and focus group. *Int J Qual Health Care* 2007;19:349–57.

16 Braun V, Clarke V, Braun, V, ClarkeV. Using thematic analysis in psychology. *Qual Res Psychol* 2006;3:77–101.

17 Haase AM, Taylor AH, Fox KR, et al. Rationale and development of the physical activity counselling intervention for a pragmatic TRial of Exercise and Depression in the UK (TREAD-UK). *Ment Health Phys Act,* 2010;3:85–91.

18 Duda JL, Williams GC, Ntoumanis N, et al. Effects of a standard provision versus an autonomy supportive exercise referral programme on physical activity, quality of life and well-being indicators: a cluster randomised controlled trial. *Int J Behav Nutr Phys Act* 2014;11:10.

19 O'Sullivan TL, Fortier MS, Faubert C, et al. Interdisciplinary physical activity counseling in primary care: a qualitative inquiry of the patient experience. *J Health Psychol* 2010;15:362–72.

20 Moore GF, Moore L, Murphy S. Facilitating adherence to physical activity: exercise professionals' experiences of the National Exercise Referral Scheme in Wales: a qualitative study. *BMC Public Health* 2011;11:935.

For peer review only

21 Dishman RK. Increasing and maintaining physical activity and exercise. *Behav Ther* 1991;22:345–78.

22 Yates BC, Anderson T, Hertzog M, et al. Effectiveness of follow-up booster sessions in improving physical status after cardiac rehabilitation: Health, behavioral, and clinical outcomes. *Appl Nurs Res* 2005;18:59–62.

23 Podlog L, Dionigi R. Psychological need fulfillment among workers in an exercise intervention: a qualitative investigation. *Res Q Exerc Sport* 2009;80:774–87.

24 Robinson H, Williams V, Curtis F, et al. Facilitators and barriers to physical activity following pulmonary rehabilitation in COPD: A systematic review of qualitative studies. NPJ Prim Care Respir Med 2018;28:19.

25 Simpson AL, Eng JJ, Tawashy AE. Exercise perceptions among people with stroke: Barriers and facilitators to participation. *Int J Ther Rehabil*;18:520-30.

For peer review only

COREQ (COnsolidated criteria for REporting Qualitative research) Checklist

A checklist of items that should be included in reports of qualitative research. You must report the page number in your manuscript where you consider each of the items listed in this checklist. If you have not included this information, either revise your manuscript accordingly before submitting or note N/A.

Topic	Item No.	Guide Questions/Description	Reported on Page No.
**Domain 1: Research team and reflexivity**			
Personal characteristics			
Interviewer/facilitator	1	Which author/s conducted the interview or focus group?	
Credentials	2	What were the researcher's credentials? E.g. PhD, MD	
Occupation	3	What was their occupation at the time of the study?	
Gender	4	Was the researcher male or female?	
Experience and training	5	What experience or training did the researcher have?	
Relationship with participants			
Relationship established	6	Was a relationship established prior to study commencement?	
Participant knowledge of the interviewer	7	What did the participants know about the researcher? e.g. personal goals, reasons for doing the research	
Interviewer characteristics	8	What characteristics were reported about the inter viewer/facilitator? e.g. Bias, assumptions, reasons and interests in the research topic	
**Domain 2: Study design**			
Theoretical framework			
Methodological orientation and Theory	9	What methodological orientation was stated to underpin the study? e.g. grounded theory, discourse analysis, ethnography, phenomenology, content analysis	
Participant selection			
Sampling	10	How were participants selected? e.g. purposive, convenience, consecutive, snowball	
Method of approach	11	How were participants approached? e.g. face-to-face, telephone, mail, email	
Sample size	12	How many participants were in the study?	
Non-participation	13	How many people refused to participate or dropped out? Reasons?	
Setting			
Setting of data collection	14	Where was the data collected? e.g. home, clinic, workplace	
Presence of non-participants	15	Was anyone else present besides the participants and researchers?	
Description of sample	16	What are the important characteristics of the sample? e.g. demographic data, date	
Data collection			
Interview guide	17	Were questions, prompts, guides provided by the authors? Was it pilot tested?	
Repeat interviews	18	Were repeat inter views carried out? If yes, how many?	
Audio/visual recording	19	Did the research use audio or visual recording to collect the data?	
Field notes	20	Were field notes made during and/or after the inter view or focus group?	
Duration	21	What was the duration of the inter views or focus group?	
Data saturation	22	Was data saturation discussed?	
Transcripts returned	23	Were transcripts returned to participants for comment and/or	

For peer review only

Topic	Item No.	Guide Questions/Description	Reported on Page No.
		correction?	
**Domain 3: analysis and findings**			
Data analysis			
Number of data coders	24	How many data coders coded the data?	
Description of the coding tree	25	Did authors provide a description of the coding tree?	
Derivation of themes	26	Were themes identified in advance or derived from the data?	
Software	27	What software, if applicable, was used to manage the data?	
Participant checking	28	Did participants provide feedback on the findings?	
Reporting			
Quotations presented	29	Were participant quotations presented to illustrate the themes/findings? Was each quotation identified? e.g. participant number	
Data and findings consistent	30	Was there consistency between the data presented and the findings?	
Clarity of major themes	31	Were major themes clearly presented in the findings?	
Clarity of minor themes	32	Is there a description of diverse cases or discussion of minor themes?	

Developed from: Tong A, Sainsbury P, Craig J. Consolidated criteria for reporting qualitative research (COREQ): a 32-item checklist for interviews and focus groups. *International Journal for Quality in Health Care*. 2007. Volume 19, Number 6: pp. 349 – 357

Once you have completed this checklist, please save a copy and upload it as part of your submission. DO NOT include this checklist as part of the main manuscript document. It must be uploaded as a separate file.

For peer review only

BMJ Open

Overcoming barriers to engagement and adherence to a home-based physical activity intervention for patients with heart failure: A qualitative focus group study

Journal:	*BMJ Open*
Manuscript ID	bmjopen-2019-036382.R2
Article Type:	Original research
Date Submitted by the Author:	20-Jul-2020
Complete List of Authors:	Okwose, Nduka; Newcastle University Faculty of Medical Sciences, Translational and Clinical Research Institute<br>O'Brien, Nicola; Northumbria University - City Campus, Department of Psychology<br>Charman, Sarah; Newcastle University Faculty of Medical Sciences, Translational and Clinical Research Institute<br>Cassidy, Sophie; Newcastle University Faculty of Medical Sciences, Translational and Clinical Research Institute<br>Brodie, David; Buckinghamshire New University, School of Health and Social Sciences<br>Bailey, Kristian; Newcastle upon Tyne Hospitals NHS Foundation Trust<br>MacGowan, Guy; Newcastle Upon Tyne Hospitals NHS Foundation Trust, Cardiology<br>Jakovljevic, Djordje; Newcastle University Faculty of Medical Sciences, Translational and Clinical Research Institute<br>Avery, Leah; Teesside University, School of Health & Life Sciences
**Primary Subject Heading**:	Cardiovascular medicine
Secondary Subject Heading:	Cardiovascular medicine, Qualitative research, Rehabilitation medicine, Sports and exercise medicine
Keywords:	Heart failure < CARDIOLOGY, QUALITATIVE RESEARCH, Adult cardiology < CARDIOLOGY

SCHOLARONE™
Manuscripts

I, the Submitting Author has the right to grant and does grant on behalf of all authors of the Work (as defined in the below author licence), an exclusive licence and/or a non-exclusive licence for contributions from authors who are: i) UK Crown employees; ii) where BMJ has agreed a CC-BY licence shall apply, and/or iii) in accordance with the terms applicable for US Federal Government officers or employees acting as part of their official duties; on a worldwide, perpetual, irrevocable, royalty-free basis to BMJ Publishing Group Ltd ("BMJ") its licensees and where the relevant Journal is co-owned by BMJ to the co-owners of the Journal, to publish the Work in this journal and any other BMJ products and to exploit all rights, as set out in our licence.

The Submitting Author accepts and understands that any supply made under these terms is made by BMJ to the Submitting Author unless you are acting as an employee on behalf of your employer or a postgraduate student of an affiliated institution which is paying any applicable article publishing charge ("APC") for Open Access articles. Where the Submitting Author wishes to make the Work available on an Open Access basis (and intends to pay the relevant APC), the terms of reuse of such Open Access shall be governed by a Creative Commons licence – details of these licences and which Creative Commons licence will apply to this Work are set out in our licence referred to above.

Other than as permitted in any relevant BMJ Author's Self Archiving Policies, I confirm this Work has not been accepted for publication elsewhere, is not being considered for publication elsewhere and does not duplicate material already published. I confirm all authors consent to publication of this Work and authorise the granting of this licence.

For peer review only

Overcoming barriers to engagement and adherence to a home-based physical activity intervention for patients with heart failure: A qualitative focus group study

Nduka C Okwose, PhD[1] , Nicola O'Brien, PhD[2,3], Sarah J Charman, PhD[1], Sophie Cassidy, PhD[1], David A Brodie[4] DSci, Kristian Bailey, MD[5] , Guy A MacGowan, MD[5,6] , Djordje G Jakovljevic, PhD[1,5,] *Leah Avery, PhD[2,6]

[1] Cardiovascular Research Centre; Translational and Clinical Research Institute, Faculty of Medical Sciences, Newcastle University, UK

[2]Population Health Sciences Research Institute, Faculty of Medical Sciences, Newcastle University, UK

[3] Department of Psychology, Northumbria University, UK

[4] School of Health and Social Sciences, Buckinghamshire New University, Uxbridge, UK

[5] Newcastle upon Tyne Hospitals NHS Foundation Trust, Newcastle upon Tyne, UK

[6]Biosciences Institute, Faculty of Medical Sciences, Newcastle University, UK

[6] School of Health and Life Sciences, Teesside University, Tees Valley, UK

Corresponding Author: Dr Leah Avery: leah.avery@tees.ac.uk, Centre for Rehabilitation, Exercise and Sports Sciences, School of Health and Life Sciences, Teesside University, Tees Valley, UK. TS1 3BA. Tel: +44 (0)1642 384130; Email: leah.avery@tees.ac.uk.

For peer review only

Abstract

Objectives: Clinical guidelines recommend regular physical activity for patients with heart failure to improve functional capacity and symptoms and to reduce hospitalisation. Cardiac rehabilitation programmes have demonstrated success in this regard, however uptake and adherence are sub-optimal. Home-based physical activity programmes have gained popularity to address these issues, although it is acknowledged that their ability to provide personalised support will impact upon their effectiveness. The study aimed to identify barriers and facilitators to engagement and adherence to a home-based physical activity programme, and to identify ways in which it could be integrated into the care pathway for patients with heart failure.

Design: A qualitative focus group study was conducted. Data were analysed using thematic analysis.

Participants: A purposive sample of 16 patients, 82% males, aged 68 ± 7 years, with heart failure duration 10 ±9 years were recruited.

Intervention: A 12 week behavioural intervention targeting physical activity was delivered once per week by telephone.

Results: Ten main themes were generated that provided a comprehensive overview of the active ingredients of the intervention in terms of engagement and adherence. Fear of undertaking physical activity was reported to be a significant barrier to engagement. Influences of family members were both barriers and facilitators to engagement and adherence. Facilitators included endorsement of the intervention by clinicians knowledgeable about physical activity in the context of heart failure; ongoing support and personalised feedback from team members, including tailoring to meet individual needs, overcome barriers and increase confidence.

For peer review only

Conclusions: Endorsement of interventions by clinicians to reduce patients' fear of undertaking physical activity and individual tailoring to overcome barriers are necessary for long-term adherence. Encouraging family members to attend consultations to address misconceptions and fear about the contraindications of physical activity in the context of heart failure should be considered for adherence, and peer-support long-term maintenance.

Strengths and limitations of this study

- Qualitative focus groups are an appropriate methodological approach to explore views on uptake, engagement and adherence to an intervention and can help facilitate consensus
- Adults with heart failure who had declined participation in the intervention and those who had previously attended and declined participation in centre-based programmes were recruited to obtain representativeness of views
- A higher proportion of men participated, therefore the views of women should be explored further
- A wide range of heart failure patients in terms of length of time since diagnosis were recruited to the study, highlighting the broad appeal of the intervention

For peer review only

Introduction

Increasing levels of physical activity is recommended to improve cardio-metabolic outcomes and to enhance overall health and psychological wellbeing[1–3]. In the UK, cardiac rehabilitation is offered as a pathway of referral for people living with chronic conditions such as cardiovascular disease. Encouraging patients to attend is often challenging, although uptake

of cardiac rehabilitation by patients following a cardiac event or procedure (e.g., MI, PCI) increased from 45% in 2013 to 50% in 2016[4]. However, these figures provide an artificial estimate of attendance in the context of heart failure, because only 5.3% of patients with heart failure actually attend cardiac rehabilitation[4]. Furthermore, dropout rates often exceed 20% for those attending centre-based programmes[5]. Barriers to uptake, engagement and adherence are reported to include proximity of cardiac rehabilitation services and motivation levels of patients to attend[5,6].

A potential solution to overcome these barriers is the provision of home-based physical activity programmes. These have shown to elicit similar outcomes to centre-based programmes and could be particularly attractive to those with a preference to increase activity levels at home. They also have the potential to be tailored to individual needs and preferences, thereby increasing engagement, adherence and reducing dropout rates[7,8].

This qualitative study explored the barriers and facilitators to engagement and adherence to a home-based physical activity intervention for patients with heart failure, called 'Active-at-Home-HF'. The findings will be used to inform optimisation of the intervention for delivery by clinicians during routine care delivery.

Methods

This study was approved by the NHS North East – Tyne and Wear South Research Ethics Committee (REC reference: 15/NE/0190). All participants provided informed written consent prior to participation.

For peer review only

Intervention

Active-at-Home-HF is a behavioural intervention designed to support patients with heart failure to increase their baseline physical activity levels by at least 2000 steps per day over a period of 12 weeks **[9].** The intervention was developed as an alternative to centre-based cardiac rehabilitation programmes to help overcome some of the well-documented barriers to engagement and adherence (e.g., travel, cost, group-based), but also as a means to support those who have previously attended centre-based programmes who wish to continue physical activity at home. Three researchers with backgrounds in exercise physiology were trained by a Chartered Health Psychologist to deliver the intervention adopting the role of a lifestyle coach. Coaches used evidence-based behaviour change techniques including behavioural goal setting, action planning, problem solving, self-monitoring, feedback on behaviour and review of behavioural goals [10, 11, 12]. Participants were supported to increase and sustain physical activity levels via telephone calls lasting between 8 and 15 minutes on at least 10 occasions, i.e. weekly over a period of 12 weeks. The number of telephone calls was dictated by the participant up to a maximum of 12 calls (i.e. one call per week). Physical activity goals were set and modified in collaboration with the coach and participant depending upon each participant's progress, capability, needs and preferences, with an emphasis on duration of physical activity rather than intensity. A standardised proforma was used to structure calls and to serve as a prompt to use all behaviour change techniques, where appropriate and to increase fidelity of delivery. All three researchers who delivered the intervention also completed study visits with partipants during the Active-at-Home-HF pilot study[13].

Design

A qualitative study embedded within a pilot study of the Active-at-Home-HF intervention was conducted [13]. The aim was to deliver the intervention to participants and obtain feedback on specific aspects of it (e.g., mode of delivery), but primarily to obtain views on whether an

For peer review only

intervention of this kind could provide the level and type of support required to increase and maintain physical activity levels. Focus group discussions were used to identify barriers and facilitators to engagement and adherence, and ways in which the intervention could be integrated into routine clinical care.

Participants

Eligible participants had left ventricular ejection fraction ≤ 40% diagnosed at least three months previously; were clinically stable and receiving an optimal medical treatment; had no contraindications to physical activity; and were able to walk and perform activities of daily living independently. Table 1 presents a summary of participant characteristics.

Table 1. Mean and SD (±) patients' demographic and clinical characteristics

Parameter	
Age (years)	67±5
Men/women	13/3
Weight (kg)	85.2±15.9
Height (m)	1.73±0.1
Duration of heart failure (years)	10±9
Medication	
ACE inhibitors	12
B- blockers	16
ARBs	3
Diuretics	9
Warfarin	5
ICD/Pacemakers	10
Comorbidities	
COPD	1
Type 2 Diabetes	5
Hypertension	16
Depression	2
Arthritis	1

For peer review only

ACE, angiotensin converting enzyme; ARB, angiotensin receptor blockers; NSAID, non-steroid anti-inflammatory drugs; COPD, chronic obstructive pulmonary disease; ICD, Implantable cardioverter defibrillator

The aim was to recruit a purposive sample of between 12 and 20 participants in line with published guidance and previously published qualitative research to ensure data saturation[13,14]. Eligible participants included those who had previously taken part and those who had refused participation in centre-based cardiac rehabilitation programme to ensure a representation of views and experiences, and also included those who had refused participation in Active-at-Home-HF to identify barriers to uptake. Participants were contacted via email, telephone, or spoken to in person to discuss participation in the study and given an opportunity to ask questions to ensure they understood the aims of the study.

Study Procedure

Eligible participants under the care of a cardiologist at the Freeman Hospital, Newcastle upon Tyne, UK were invited to take part in the study. Focus group discussions were facilitated by two female Chartered Health Psychologists with expertise in health behaviour change, physical activity intervention and qualitative research methods. Neither of the researchers had met the participants prior to the focus group discussions and neither were aware of any preliminary findings of the Active-at-Home-HF pilot study. The Active-at-Home-HF behavioural intervention was developed by one of the Health Psychologists conducting the focus groups. The focus group discussions were conducted in a meeting room at the Royal Victoria Infirmary, Newcastle upon Tyne. Only the two researchers and the participants were present during the discussions. One of the researchers explained the aims and objectives of the focus group discussions to set the scene, and the role of both psychologists within the research team. This included how the data generated from the focus group discussions would be used, i.e. their goal

For peer review only

was to use the findings to further develop the intervention and/or integrate it within usual clinical care to help other people with heart failure.

Materials

The research team developed a topic guide to structure discussions. Topics included reasons for taking part, or declining participation; preference for completing a home-based programme; information required to inform decision-making regarding participation; support requirements to adhere to and complete the programme and potential barriers; and expectations of the programme. All questions were open-ended and prompts were used to generate a deeper understanding of participants' views. Each focus group discussion was audio recorded and transcribed verbatim.

Methodological Quality and reporting

The research was conducted in accordance with the consolidated criteria for reporting qualitative research (COREQ) to enhance transparency and maximise methodological quality[15]. To reduce bias from responders, members of the team delivering the intervention or involved in the pilot study clinic visits were not involved with focus group discussions.

Data Analysis

Data were analysed by hand using thematic analysis[16]. To maximise methodological quality and trustworthiness of the findings, the following analysis procedure was undertaken: (i) Two researchers independently read and re-read focus group transcripts (NCO and LA); (ii) both researchers independently applied codes to segments of data within the first focus group transcript to develop initial themes and sub-themes; (iii) the same two researchers discussed findings and agreed a preliminary set of themes and sub-themes; (iv) one researcher (NCO) repeated this process with the remaining two transcripts; and (v) both researchers agreed the final set of themes and sub-themes that adequately represented the data set following

For peer review only

discussion. Supporting direct quotes from participants were subsequently applied to themes and sub-themes. A third researcher (NO'B) reviewed themes and sub-themes generated to verify interpretation. We did not consider it necessary to use qualitative data analysis software due to the small number of focus group transcripts. The aim was to avoid deterministic processes, which can be a risk with qualitative software, and to prioritise gaining an in depth understanding of the data generated. We achieved this with in depth scrutiny of the transcripts by hand.

Patient and public involvement

There was no patient and public involvement in the design or planning of the study.

Results

Three focus groups were conducted, with a duration of 47, 53 and 83 minutes respectively. A total of sixteen individuals (13 males, 3 females; median age 68 years; interquartile range: 6.5 years) from the twenty who were eligible to take part in the pilot study, and two who were eligible but declined participation took part in a focus group discussion. Participants had either completed Active-at-Home-HF (4 participants), were still participating at the time of the focus group (10 participants), or had declined participation (2 participants). Three participants had previously declined centre-based cardiac rehabilitation. Ten participants had implanted cardiac defibrillators (ICDs). Fifteen of the sixteen participants were retired from active work. Following thematic analyses, 10 main themes were generated. It was noted following the conduct of focus group three that no new data were generated, i.e. it was agreed that data saturation was reached. Table 2 provides a summary of the themes and subthemes, and example direct quotes to provide context.

For peer review only

Table 2: A summary of themes and subthemes derived from thematic analyses of focus group discussion transcripts

Theme	Sub-theme	Quotes
**1. Fear of undertaking physical activity**		'I'm not confident when I go out on my own. I'm frightened I'm going to collapse' (FG 1, Male, aged 57)<br><br>'It's frightening when you've gone too far. Something's always holding me back from going that little bit further, you know' (FG 1, Male, aged 56)
**2. Family members influence physical activity efforts**	**2.1 Fear in family members prevents engaging with physical activity**	'I've got a wife who's not happy if I say I'm going out for a walk for an hour.., because she thinks that's too much' (FG 2, Male, aged 67)<br><br>'..Sometimes, your family don't want you to do it, not because they're being horrible, it's because they're concerned about you' (FG 1, Male, aged 78)
	**2.2 Support from family members facilitates physical activity**	'I needed motivation which I got from the family, which is great…I don't know how I would have fared without family' (FG 2, Female, aged 62)<br><br>'I think it does help if you've got a supportive partner. My husband has come out with me for all my walks right from the start. I think it's good if your partners can be involved and then they realise, don't they, how they can help' (FG 2, Female, aged 62)
**3. Physical activity programmes require endorsement by clinicians**		'I think my biggest breakthrough was . . . I was doing more and more but, again, family was saying, "Take it easy." Fortunately, my wife was with me on one occasion when I saw Dr "XX" and he asked what I'd been doing, and he said, "Well, do more." I said,

For peer review only

	“How do you mean by that?” He said, “Just do more and more until you feel you can’t and then back off a bit.” So, fortunately, my wife was there and she heard him say it. So, I’ve had no peace since then’ (FG 1, Male, aged 78)<br><br>‘My nurse certainly was interested [in my physical activity habits] and when I went last time, was aware of what I was doing and obviously encouraging’ (FG 2, Female, aged 62)
**4. Completion of a cardiac rehabilitation programme provides confidence to complete a physical activity programme at home**	‘I think the most important thing when you've had a heart attack is to have the confidence to do it at all. I think the cardiac rehabilitation that I had gave me the confidence and then you can move on to do other things. I think if you had just had a heart attack and someone says, "Right, you've got to go for a walk every day," it would be difficult to do. I think it would. So it's like a step in the process’ (FG 2, Male, aged 71)<br><br>‘I was really upset when it [cardiac rehabilitation] finished. I had somewhere to go. I’m sat at home by myself all day, so coming here on the Wednesday [for the study visit] and receiving feedback, it was like Christmas all over again, you know. It was great. (FG 1, Male, aged 56)
**5. Coach support increases motivation long-term**	‘[having your coach call you] encourages you to keep up with it, definitely, because I think if you didn't get the phone calls, I think you might just go, "It's not very nice out today, I'll not bother going’(FG 2, Female, aged 62)

For peer review only

	'When somebody is monitoring you, it makes you get up and go out, doesn't it? I mean if you go to the gym yourself, some mornings you might say, "I'm not going to go today," but it's helping you, isn't it?' (FG 3, Male, aged 62)
**6. Weekly agreed targets increased confidence and motivation**	'It's a target as everyone's saying and you want to do it and you feel very enthusiastic about doing it and I will certainly continue after the 12 weeks because it would be pointless stopping all together wouldn't it? It would waste the benefit sort of thing' (FG 2, Male, aged 70)<br><br>'I think if you were told increase to 12,000 and I come back in 12 weeks' time and we talk about it, you would go... but what's good is on Tuesday [my coach] rings me and goes through all the information, we get an average and then he'll ask me how I'm feeling, etc., etc., and say, "Right, let's try and take that 12,000 up to 12,500." That's on a weekly basis. You know for a fact that someone's interested in what's been happening for the past week and we can take that from where we are now to try and improve things.' (FG 2, Male, aged 69)
**7. A credible team increased the likelihood of participation**	'I think the fact that people like yourselves who are specialists in this sort of area take such an interest in us people. I think that gives you the boost again. I think it does boost people when you've got people who are really even higher than your GP and what not in that specific area of cardiac problems' (FG 2, Male, aged 78)

For peer review only

	'Well I think this is a speciality subject, what you're doing and all the stuff here that we've been doing. I think it's better in that situation to get the right advice' (FG 2, Male, aged 67)
**8. The surrounding environment creates barriers to increased physical activity/exercise**	'I think another …. difficulty… trying to get people motivated is the area that they live in. Now if you've got a bit of countryside, open fields and that, it opens you to more space unlike just got solid concrete, trying to get people motivated to walk down the same street or go a particular… way, that's going to be difficult I think. If you're going to motivate people you're going to have to think where they actually live because people need different types of motivation. For people who live near the coast and the countryside, people living smack bang in the middle of a built-up area etc' (FG 2, Male, aged 67)<br>'To me, I'm okay most of the time on the flat, but it's any incline. The littlest incline in the world kills me' (FG 2, Male, aged 78)<br>'I think some of the benefit was the climate as well, it's a struggle in wind and rain' (FG 3, Female, aged 63)
**9. Participation prompts an increase in every day activity levels**	'What I do now is I have to drive to the supermarket but I park in the furthest corner of the car park and I walk round the car park. Then on rainy days, what I have been doing is going into the supermarket and going round it twice before I start my shopping. The people must think you're mad if they look at you on the CCTV' (FG 2, Male, aged 71)

For peer review only

		'I think it's got to work doing exercise. I mean they brought me in here; they're going on about putting a defibrillator in. Now when he saw me, how my heart had changed just through doing things, he decided he wouldn't put one in' (FG 3, Male, aged 62)<br><br>'I haven't been back to see my specialist at the Freeman [hospital], because he doesn't want to see me for 12 months because I'm too fit for him. I said, "Are you sick of me?" he said, "No." It was six months before and then I went for the last check sometime last year, he said, "Right, I don't want to see you for 12 months. I said, have you gone off me?' (FG 2, Male, aged 71)<br><br>'Well since I've completed the course [Active-at-Home-HF], I've been diabetic for 30 years, my blood sugar levels have never been as normal as an ordinary person's in my life' (FG 2, Male, aged 79)
**10. Support to maintain long-term activity levels would be beneficial**	**10.1 Support from a healthcare professional following completion of the programme would help maintain increased physical activity**	'I think a follow-up is a good idea. You need some sort of follow-up after you've finished. How they do it, whether it's a phone call or a meeting with your doctor or whatever, your GP or anybody like that, I don't know how they would do it. But I think that's quite important that. Then you wouldn't feel as if you've been chucked on the scrap heap type of thing, you've finished, it's done' (FG 3, Male, aged 73)<br><br>'Do you know, that's the worst thing, when you've finished your course and the following Wednesday there's no phone call. It's horrendous isn't it?' (FG 2, Male, aged 67)

For peer review only

**10.2 Group peer support would promote long-term physical activity**	'I know it's just the start of this programme …, As far as feedback on it is concerned, maybe something about halfway through, maybe six weeks you could have a little meeting with some of the people just for half an hour, just have a little chat and see who's there, what's what, talk to people, something like that anyway. A little informal meeting or a social evening or whatever you want to call it. It lets everybody else know that it's not just you or another two or three people, it might be 20 people' (FG 3, Male, aged 73)

For peer review only

Fear of undertaking physical activity

Once diagnosed with heart failure, participants reported difficulties overcoming the fear associated with the diagnosis (i.e. future cardiac events and overexertion) and increasing physical activity exacerbated this fear. While some reported that they could still engage in some physical activity, there was concern that too much would be detrimental. This was reported to be a significant barrier to participation: *'It's frightening when you've gone too far. Something's always holding me back from going that little bit further, you know'* (FG 1, Male, aged 56). In this regard, it was reported that reassurance from a clinican is required to confirm that increasing physical activity is safe, and specific information in terms of how much activity should be undertaken to be beneficial, and not detrimental.

Family members influence engagement with physical activity

It was reported that family members were both a barrier and facilitator to engaging in physical activity. Some participants reported that family members dissuaded them from engaging in physical activity. Although, there was a consensus that this was linked to fear and concern *'Sometimes, your family don't want you to do it, not because they're being horrible, it's because they're concerned about you'* (FG 2, Male, aged 78). Fear of family members often meant that participants were less active following a diagnosis of heart failure than they were previously.

It was also reported that family members were a strong motivating factor once they were reassured that increasing physical activity was beneficial. For some participants, having a partner or relative to support them and participate in new activity routines was reported as a significant facilitator to continued participation. A typical quote included: *'I think it does help if you've got a supportive partner. My husband has come out with me for all my walks right*

For peer review only

from the start. I think it's good if your partners can be involved and then they realise, don't they, how they can help' (FG 2, Female, aged 62). There was a consensus that without the practical and emotional support of partners and relatives, increasing physical activity would be more difficult.

Physical activity programmes require endorsement by clinicians

Endorsement of the physical activity programme by a clinician was reported to reduce patients' fears about increasing levels of physical activity and potentially making their condition worse. Furthermore, it reassured family members when they witnessed clinicians explaining the benefits and advising an increase. In addition, ongoing positive reinforcement by the clinical team was considered paramount to continued participation: '*My nurse certainly was interested [in my physical activity levels] and when I went last time, was aware of what I was doing and was encouraging'* (FG 2, Female, aged 62).

Prior completion of a cardiac rehabilitation programme provides confidence to engage with home-based physical activity programmes

Cardiac rehabilitation was reported to play a significant role in confidence building among heart failure patients, including confidence to continue with activities of daily living. As such it was reported to be a significant facilitator to uptake of Active-At-Home-HF as a means of providing patients with ongoing support and encouragement to be physically active in the long-term. Continued participation and feedback was particularly important to those who had completed a cardiac rehabilitation programme: *'I was really upset when it [cardiac rehabilitation] finished. I had somewhere to go. I'm sat at home by myself all day, so coming here on the Wednesday [for the study visit] and receive feedback, it was like Christmas all over*

For peer review only

again, you know. It was great' (FG 1, Male, aged 56). This quote highlights the importance of social support and feedback in order to promote uptake and adherence to cardiac rehabilitation. Both were provided via Active-at-Home-HF and were reported to be essential.

Behavioural support provided by a trained professional increases motivation and adherence

Participants reported that having a trained professional who monitored their performance and provided personalised advice and support promoted continued participation (i.e. adherence): *'[having your coach call you] encourages you to keep up with it, definitely, because I think if you didn't get the phone calls, I think you might just go, "It's not very nice out today, I'll not bother going'* (FG 2, Female, aged 62). It was important that the support was provided by someone who could advise on the type, amount and duration of activity required to reach a specific target; and someone who was skilled in the use of behavioural strategies who could appropriately challenge participants and support them to overcome perceived barriers.

Agreed weekly targets increased confidence and motivation

Physical activity goal setting was considered an important mechanism to increase motivation, particularly when weekly goals were personalised and agreed between the participant and coach. This provided reassurance that goals were realistic, attainable and safe and it was reported to be rewarding when goals were achieved. Some participants were keen to set themselves daily or weekly targets once they had knowledge and confidence in their capabilities, highlighting the potential long-term impact of the programme.

For peer review only

'It's a target as everyone's saying and you want to do it and you feel very enthusiastic about doing it and I will certainly continue after the 12 weeks because it would be pointless stopping all together wouldn't it?' (FG 2, male, aged 70).

A credible team increases the likelihood of participation

An incentive of taking part in Active-at-Home-HF was reported to be the support and monitoring offered by experts in heart failure and physical activity. Participants emphasised that endorsement of the programme by a Consultant who was knowledgeable about the benefits of physical activity in the context of heart failure, and a referral to a trained professional/member of his or her team who was aware of their clinical circumstances helped overcome barriers to ongoing participation. The 'knowledgeable team' provided reassurance around safety, and was skilled in tailoring the intervention to individual needs and preferences and this was considered important to facilitate initial engagement and adherence.

'Well I think this is a speciality subject, what you're doing and all the staff here know what we've been doing. I think it's better in that situation to get the right advice' (FG 2, Male, aged 67).

The surrounding environment creates barriers to increasing physical activity and maintaining a physically active lifestyle

Environmental factors including living in hilly areas or in surroundings that were not considered picturesque were reported to be barriers to engagement and ongoing participation in physical activity. Poor weather conditions were most frequently reported to impact negatively on motivation and long-term participation.

For peer review only

'To me, I'm okay most of the time on the flat, but it's any incline. The littlest incline in the world kills me' (FG 2, Male, aged 78).

'I think some of the benefit was the climate as well, because it was through the summer' (FG 3, Female, aged 63).

Participants reported that the support they received via Active-at-Home-HF enabled them to identify ways in which they could increase their levels of activity despite these barriers, for example planning alternative ways to increase their step count and reach their overall goals. In this regard the programme increased motivation and realisation that there was an alternative approach to increasing activity to a level sufficient to have a clinically meaningful impact.

Participation prompts an increase in every day activity levels and leads to unexpected positive outcomes

Active-at-Home-HF was reported to encourage participants to seek ways to increase their activity levels by modifying their everyday behaviours (e.g. walking instead of driving). One participant reported how the intervention prompted him to spend more time with his grandchildren, thereby positively affecting activity levels and social connections with his family. Some described how their fitness and feeling of wellbeing had improved (this was confirmed in the pilot study) and the frequency of appointments with their consultant had reduced following progress.

'I haven't been back to see my specialist at the hospital, because he doesn't want to see me for 12 months because I'm too fit for him. I said, "Are you sick of me?" he said, "No." It was six months before and then I went for the last check sometime last year, he said, "Right, I don't want to see you for 12 months' (FG 2, male, aged 71).

For peer review only

The intervention led to improvements in cardiac function for the majority of those who participated[9]. For some, this outcome meant avoiding invasive procedures that were planned prior to the study. For other participants the intervention helped to improve cardiac function and control other co-morbidities[9].

> *'Well since I've completed the course [Active-at-Home-HF], I've been diabetic for 30 years, my blood sugar levels have never been as normal as an ordinary person's in my life'* (FG 2, male, aged 79).

Ongoing support would facilitate long-term maintenance of physical activity

Continued support was considered critically important for psychological and emotional wellbeing as well as programme adherence and maintenance of activity levels following completion of the intervention. It was emphasised that support should include a review of progress to promote ongoing motivation.

> *'Do you know, that's the worst thing, when you've finished your course and the following Wednesday there's no phone call. It's horrendous isn't it?'* (FG 2, male, aged 67).

There was a general consensus that having meetings with others who have taken part in the programme would be beneficial for social support and promoting increased physical activity levels long-term.

> *'Maybe something about halfway through, maybe six weeks you could have a little meeting with some of the people just for half an hour, just have a little chat and see who's there, what's what, talk to people, something like that anyway. A little informal meeting or a social evening or whatever you want to call it. It lets everybody else know*

For peer review only

that it's not just you or another two or three people, it might be 20 people' (FG 3, male, aged 73).

A consistent theme across focus group discussions was the need for ongoing support to maintain physical activity levels and the clinical benefit observed. Participants reported the omission of this from other programmes they had taken part in, including cardiac rehabilitation. It was acknowledged that provision of support wasn't necessarily the role of the consultant, however it was important to have a trained team member who was motivated to support patients to increase and maintain their activity levels and who was knowledgeable in this area. Peer-support was considered important for long-term maintenance of physical activity following completion of the programme as a means of continuing activity consistently and safely.

Discussion

Although physical activity is widely advocated to improve health, it is viewed as a significant challenge for people living with chronic health conditions and regularly increases fear. Fear of increasing physical activity was reported in the present study and as such, the need for positive reinforcement and reassurance from clinicians who are advocates of physical activity in the context of heart failure was considered necessary to overcome this barrier. Family members and partners were reported to reinforce fear, and restricted activity levels of participants for fear of an adverse event occurring. Involvement of significant others in consultations could provide a solution to this problem and should be explored further.

Participating in cardiac rehabilitation was reported to reduce psychological stress and fear associated with physical activity and was a facilitator to uptake of Active-at-Home-HF. This finding was further substantiated by the sample of participants taking part in the study (i.e., it included those who had previously completed centre-based cardiac rehabilitation who were keen to continue). Just like community or clinic based cardiac rehabilitation, it is important for

For peer review only

home-based interventions to be holistic where possible[17]. Duda and colleagues reported that the success of an exercise intervention depended upon the intensity and type of support offered[18]. Active-at-Home-HF involved the use of lifestyle coaches who were trained to use specific evidence-based behaviour change techniques to target increased physical activity, and this training was essential to provide the necessary skills and confidence to effectively deliver the intervention. Although coaches, through their academic training, were able to communicate the physiological and health benefits of increased physical activity in the context of heart failure, which is important for motivation/engagement[19] and adherence[20], they reported that without behavioural training they would have struggled specifically to support participants to overcome barriers and this would have impacted upon adherence. This suggests that training of those required to deliver the intervention is critically important to support enactment and maintenance of physical activity. Therefore, behavioural training and the provision of a standardised proforma were considered facilitators to intervention delivery.

The need to involve clinicians to advocate and support increased physical activity at home was considered vital to reassure patients about safety and efficacy, to add credibility and to successfully integrate the programme in to routine clinical care. In this regard participants were more likely to participate and adhere to the programme if a clinician had advocated it and were made aware of their progress. A potential limitation of our study is that only two individuals who declined participation in Active-at-Home-HF took part in a focus group discussion. This provides limited insights in terms of barriers to uptake and this should be explored further. Although, encouragingly, 25% of our sample had previously declined participation in centre-based cardiac rehabilitation programmes. Our findings suggest that the decision to participate in Active-at-Home-HF was largely due to the way in which it was offered to patients by clinicians who were positive about the programme, and who could provide reassurance about its safety and overall benefits. This is further reflected in our sample, which included

For peer review only

participants who had been diagnosed with heart failure for an average of ten years (SD +/- 9 years), which could be considered unusual (i.e. it may have been expected that those newly diagnosed would be more likely to engage). However, our sample included those who had previously declined cardiac rehabilitation, those who had attended, and those who had a long history of heart failure who had never been offered rehabilitation, demonstrating the flexibility of the intervention and it acceptance to a broad range of patients.

Currently, attendance at cardiac rehabilitation in hospitals or community centres involves frequent travel and associated costs, and these are barriers to participation, especially for those who have limited financial resources or who are unable to drive. Furthermore, for some patients a group-based environment reduces confidence and doesn't always provide the one-to-one support they require. Active-at-Home-HF is an alternative to help overcome these barriers while providing patients with a personalised programme capable of increasing activity levels and impacting positively on health and fitness.

Previous studies support the use of supportive calls as an effective way to motivate patients to maintain physical activity levels, particularly those who have a low level of physical functioning[21]. The Active-at-Home-HF intervention was delivered by short telephone calls and this proved to be an effective way of targeting and maintaining increased physical activity while providing support. The brief nature of these telephone interactions increases the likelihood that this intervention could be feasibly rolled out within routine clinical practice. However, our training programme focussed only on the delivery of behavioural strategies by coaches who were already knowledgeable about the role of physical activity in the context of heart failure. Therefore, this suggests the potential need for training developed specifically for clinicians to ensure they have the knowledge and skills required to effectively deliver all aspects of the intervention. A commonly suggested facilitator to ongoing engagement and maintenance of physical activity levels following completion of the programme was having an

For peer review only

opportunity to meet with others who had completed the programme as a means of social support. This is an important consideration with 40-50% of those participating in physical activity programmes relapsing and returning to their previous physical activity states within six months[21,22]. Such groups provide a strong form of social support where long-term friendships are developed that are essential for long-term engagement and encouragement[20]. Furthermore, these groups bring together individuals with similar conditions and work to generate a sense of obligation thereby facilitating adherence and this was highlighted by participants[23]. It is important to highlight that group support was suggested mainly at the post intervention time-point to promote maintenance. This reinforces the need for some to gain mastery and confidence around increasing their activity levels before entering a group environment.

To the best of our knowledge, this is the first qualitative study to explore barriers and facilitators to participation and adherence to a home-based physical activity programme in the context of heart failure. In addition, we identified ways in which the intervention could be successfully integrated within routine clinical care. Our findings highlight that facilitators to home-based physical activity programmes include endorsement by members of the clinical team, where possible a consultant, and that clinicians should be advocates of and knowledgeable about physical activity in the context of heart failure. While it is acknowledged that not all clinical teams will have the knowledge, expertise and skills to effectively promote physical activity, the findings from our study suggest that a future intervention should incorporate training for clinicians to address this issue, and this would help facilitate future roll-out. Those who declined participation in Active-at-Home-HF did so out of fear and lack of knowledge about the specific benefits of physical activity in the context of heart failure. Specifically, participants believed that physical activity wouldn't improve their condition and fear of a cardiac event outweighed any possible benefits of an intervention. Findings also

For peer review only

suggested that partners/family members reinforced fear and beliefs about efficacy. This suggests that a future intervention should incorporate specific learning/education content about the role of physical activity in addition to a recommendation from a clinical team member, and involve family members, where possible to overcome misconceptions. Participants were supported throughout the duration of the intervention by research team members with backgrounds in clinical exercise physiology who were trained by a Health Psychologist to use evidence-based behaviour change techniques to target increased physical activity and maintenance over time. Participants reported this support as essential to obtain feedback, maintain motivation, to challenge their perceptions and it was beneficial as a form of social support. Therefore a future intervention should involve training of clinical team members to provide this support to facilitate full integration of the intervention in routine clinical care.

In summary, the most salient facilitators to uptake and engagement of the Active-at-Home-HF intervention were: clinical team members who provided personalised information about the benefits and safety of the intervention, and involvement of family members during consultations to provide them with reassurance and encourage them to provide support. In the context of health behaviour change theory, this approach targets attitudes, beliefs, risk perceptions and self-efficacy of patients that increases the likelihood of intention formation (i.e. engagement with the programme). Facilitators to adherence included behavioural prompting (i.e. a telephone call) by a knowledgeable team member who can provide individualised feedback and support to enable patients to set and revise activity goals, and identify ways to overcome barriers to reaching these goals. In combination these self-regulatory strategies target adherence and support maintenance of physical activity. Positive reinforcement from a consultant (e.g., positive feedback on increased activity levels in relation to outcomes) was considered a further facilitator to adherence. Although involvement of family members wasn't a formal component of the Active-at-Home intervention, in instances where

For peer review only

partipants did involve family members in their consultations they reported this to be of significant benefit to adherence. Future delivery of an optimised version of this intervention should consider uptake, engagement and adherence separately and use the facilitators identified to target each.

Although it's possible that patients referred to rehabilitation with other long-term health conditions can present with unique challenges that create barriers to uptake, engagement and adherence, the findings of our qualitative study revealed parallels with other clinical groups. A 2018 systematic review of qualitative studies in the context of COPD reported fear and lack of self-efficacy as barriers to uptake and engagement, and the relationship with healthcare professionals, personalised feedback, and peer interaction as facilitators to engagement and adherence[24]. Similarly in the context of stroke, barriers and facilitators included fear of a subsequent stroke, and personalised feedback and support to promote adherence[25]. These findings suggest that commonalities exist, and the way in which we communicate and support patients to increase and maintain physical activity levels is vitally important. As such there is potential to adapt the Active-at-Home-HF intervention for delivery in a range of contexts.

Funding

This research was funded by the National Institute for Health Research Biomedical Research Centre (grant number: BH142109). The funder and study sponsor played no role in the study design; collection, analysis and interpretation of data; writing the manuscript; or the decision to submit the manuscript for publication.

Competing Interest

The authors declare that they have no competing interests

Author Contributions

For peer review only

LA, DJ, and NCO conceived the study and its design. LA supervised its conduct. KB and GM facilitated recruitment of participants and collected demographic data. SJC, SC and NCO delivered the Active-at-Home-HF intervention and conducted study visits that involved data collection. LA and NO'B conducted all focus group discussions; NCO, LA and NO'B analysed and interpreted data; NCO and LA drafted the manuscript. All authors LA, NCO, DJ, DB, KB, GM, SJC, SC and NO'B made an important intellectual contribution, revised the manuscript and approved the version to be published. LA is accountable for all aspects of the study, including the accuracy and integrity of the data.

Acknowledgements

The authors would like to acknowledge and thank the participants who took part in this study.

Data sharing statement

Participants were not asked to provide consent to share the transcripts of their focus group discussion beyond the research team.

References

1 O'Donovan G, Hillsdon M, Ukoumunne OC, et al. Objectively measured physical activity, cardiorespiratory fitness and cardiometabolic risk factors in the Health Survey for England. *Prev Med* 2013;57:201–5.

2 Colberg SR. Physical activity: The forgotten tool for type 2 diabetes management. *Front Endocrinol* 2012;3:70.

3 Josefsson T, Lindwall M, Archer T. Physical exercise intervention in depressive disorders: Meta-analysis and systematic review. *Scand J Med Sci Sports* 2014; 24:259-

72.

4 British Association for Cardiovascular Prevention and Rehabilitation. National Audit of Cardiac Rehabilitation: Annual Statistical Report 2017. *Annu Rep* 2017;:1–72.https://www.bhf.org.uk/publications/statistics/national-audit-of-cardiac-rehabilitation-annual-statistical-report-2017

5 Piotrowicz E, Baranowski R, Bilinska M, et al. A new model of home-based telemonitored cardiac rehabilitation in patients with heart failure: Effectiveness, quality of life, and adherence. *Eur J Heart Fail* 2010;12:164–71.

6 Jolly K, Taylor RS, Lip GYH, et al. A randomized trial of the addition of home-based exercise to specialist heart failure nurse care: The Birmingham Rehabilitation Uptake Maximisation study for patients with Congestive Heart Failure (BRUM-CHF) study. *Eur J Heart Fail* 2009;11:205–13.

7 Zwisler A-D, Norton RJ, Dean SG, et al. Home-based cardiac rehabilitation for people with heart failure: A systematic review and meta-analysis. *Int J Cardiol* 2016;221:963–9.

8 Oerkild B, Frederiksen M, Hansen JF, *et al.* Home-based cardiac rehabilitation is as effective as centre-based cardiac rehabilitation among elderly with coronary heart disease: Results from a randomised clinical trial. *Age Ageing* 2011;40:78–85.

9 Okwose N, Avery L, O'Brien N, et al. Acceptability, Feasibility and Preliminary Evaluation of a Novel, Personalised, Home based Physical Activity Intervention for Chronic Heart Failure (Active-at-Home-HF): A Pilot Study. *Sports Med Open*, 2019; 5: 45.

10 Avery L, Flynn D, Van Wersch A, et al. Changing physical activity behavior in type 2

For peer review only

diabetes: A systematic review and meta-analysis of behavioral interventions. *Diabetes Care*. 2012;35:2681–9.

11 Avery L, Flynn D, Dombrowski SU, et al. Successful behavioural strategies to increase physical activity and improve glucose control in adults with Type 2 diabetes. *Diabet Med*. 2015;32:1058–62

12 Avery L, Charman SJ, Taylor L et al. Systematic development of a theory-informed multifaceted behavioural intervention to increase physical activity of adults with Type 2 diabetes in routine primary care: Movement as Medicine for Type 2 Diabetes, Imp Sci,11,99.

13 Saunders B, Sim J, Kingstone T, et al. Saturation in qualitative research: exploring its conceptualization and operationalization. *Qual Quant* 2018;52:1893–907.

14 Avery L, Exley C, McPherson S, et al. Lifestyle Behavior Change in Patients With Nonalcoholic Fatty Liver Disease: A Qualitative Study of Clinical Practice. *Clin Gastroenterol Hepatol* 2017;15:1968–71.

15 Tong A, Sainsbury P, Craig J. Consolidated criteria for reporting qualitative research (COREQ): a 32- item checklist for interviews and focus group. *Int J Qual Health Care* 2007;19:349–57.

16 Braun V, Clarke V, Braun, V, ClarkeV. Using thematic analysis in psychology. *Qual Res Psychol* 2006;3:77–101.

17 Haase AM, Taylor AH, Fox KR, et al. Rationale and development of the physical activity counselling intervention for a pragmatic TRial of Exercise and Depression in the UK (TREAD-UK). *Ment Health Phys Act,* 2010;3:85–91.

18 Duda JL, Williams GC, Ntoumanis N, et al. Effects of a standard provision versus an

For peer review only

autonomy supportive exercise referral programme on physical activity, quality of life and well-being indicators: a cluster randomised controlled trial. *Int J Behav Nutr Phys Act* 2014;11:10.

19 O'Sullivan TL, Fortier MS, Faubert C, et al. Interdisciplinary physical activity counseling in primary care: a qualitative inquiry of the patient experience. *J Health Psychol* 2010;15:362–72.

20 Moore GF, Moore L, Murphy S. Facilitating adherence to physical activity: exercise professionals' experiences of the National Exercise Referral Scheme in Wales: a qualitative study. *BMC Public Health* 2011;11:935.

21 Dishman RK. Increasing and maintaining physical activity and exercise. *Behav Ther* 1991;22:345–78.

22 Yates BC, Anderson T, Hertzog M, et al. Effectiveness of follow-up booster sessions in improving physical status after cardiac rehabilitation: Health, behavioral, and clinical outcomes. *Appl Nurs Res* 2005;18:59–62.

23 Podlog L, Dionigi R. Psychological need fulfillment among workers in an exercise intervention: a qualitative investigation. *Res Q Exerc Sport* 2009;80:774–87.

24 Robinson H, Williams V, Curtis F, et al. Facilitators and barriers to physical activity following pulmonary rehabilitation in COPD: A systematic review of qualitative studies. NPJ Prim Care Respir Med 2018;28:19.

25 Simpson AL, Eng JJ, Tawashy AE. Exercise perceptions among people with stroke: Barriers and facilitators to participation. *Int J Ther Rehabil*;18:520-30.

For peer review only
